# Binding Specificity of Native Odorant-Binding Protein Isoforms Is Driven by Phosphorylation and *O*-N-Acetylglucosaminylation in the Pig *Sus scrofa*

**DOI:** 10.3389/fendo.2018.00816

**Published:** 2019-01-25

**Authors:** Patricia Nagnan-Le Meillour, Alexandre Joly, Chrystelle Le Danvic, Arul Marie, Séverine Zirah, Jean-Paul Cornard

**Affiliations:** ^1^Unité de Glycobiologie Structurale et Fonctionnelle, UMR8576, USC-UGSF INRA 1409, CNRS-Université de Lille, Lille, France; ^2^ALLICE R&D, Paris, France; ^3^Unité Molécules de Communication et Adaptation des Microorganismes, Muséum National d'Histoire Naturelle, UMR 7245 CNRS/MNHN, Paris, France; ^4^Laboratoire de Spectroscopie Infrarouge et Raman, UMR8516 CNRS-Université de Lille, Lille, France

**Keywords:** *O*-GlcNAc, odorant-binding protein, phosphorylation, CID-nano-LC-MS/MS, fluorescence spectroscopy, pheromone

## Abstract

Odorant-binding proteins (OBP) are secreted in the nasal mucus at the vicinity of olfactory receptors (ORs). They act, at least, as an interface between hydrophobic and volatile odorant molecules and the hydrophilic medium bathing the ORs. They have also been hypothesized to be part of the molecular coding of odors and pheromones, by forming specific complexes with odorant molecules that could ultimately stimulate ORs to trigger the olfactory transduction cascade. In a previous study, we have evidenced that pig olfactory secretome was composed of numerous olfactory binding protein isoforms, generated by *O*-GlcNAcylation and phosphorylation. In addition, we have shown that recombinant OBP (*stricto sensu*) produced in yeast is made up of a mixture of isoforms that differ in their phosphorylation pattern, which in turn determines binding specificity. Taking advantage of the high amount of OBP secreted by a single animal, we performed a similar study, under exactly the same experimental conditions, on native isoforms isolated from pig, *Sus scrofa*, nasal tissue. Four fractions were obtained by using strong anion exchange HPLC. Mapping of phosphorylation and *O*-GlcNAcylation sites by CID-nanoLC-MS/MS allowed unambiguous localization of phosphosites at S13 and T122 and HexNAc sites at S13 and S19. T112 or T115 could also be phosphorylated. BEMAD analysis suggested extra phosphosites located at S23, S24, S41, S49, S57, S67, and T71. Due to the very low stoichiometry of GlcNAc-peptides and phosphopeptides, these sites were identified on total mixture of OBP isoforms instead of HPLC-purified OBP isoforms. Nevertheless, binding properties of native OBP isoforms to specific ligands in *S. scrofa* were monitored by fluorescence spectroscopy. Recombinant phosphorylated OBP-Pichia isoforms bind steroids and fatty acids with slight differences. Native isoforms, that are phosphorylated but also *O*-GlcNAcylated show radically different binding affinities for the same compounds, which strongly suggests that *O*-GlcNAcylation increases the binding specificity of OBP isoforms. These findings extend the role of *O*-GlcNAc in regulating the function of proteins involved in many mechanisms of metabolic homeostasis, including extracellular signaling in olfaction. Data is available via ProteomeXChange with identifier PXD011371.

## Introduction

In olfaction, one major challenge is to understand how odors are encoded in the nasal cavity by molecular players, olfactory receptors (OR) and odorant-binding proteins (OBP). Indeed, since their identification 30 years ago [(1); Nobel Price 2004], most of the OR are still orphan, as their ligands have not been identified. OBPs are secreted by the cells of Bowman's gland in the nasal mucus where dendrites of olfactory receptor neurons containing ORs are bathing (2, 3). OBPs are supposed to at least, act as an interface between airborne odorants and aqueous medium containing ORs. While OBPs binding to odors is well-documented at the biochemical level, very few investigations into OR binding have been reported. Activation of ORs with odorant molecules alone, with OBP bound or unbound to odorants, have been measured by electrophysiology [e.g., (4)] or other indirect techniques [e.g., (5, 6)]. This lack of direct evidence explains why the nature of ORs ligands is still uncertain. In mammals, OBPs have been suggested to form a specific complex with a given odorant that could specifically interact with an OR, leading to the initiation of the olfactory transduction cascade. Such a molecular coding mechanism requests an equal diversity in odorant molecules, OBPs and ORs. In each mammalian species, only a few genes (2–8) encode OBPs, whilst the OR family comprises around 1,000 genes (1), and the number of odorants is theoretically unlimited.

In previous work (7–9), we have evidenced the diversity of OBPs from pig olfactory secretome, which is generated by phosphorylation and/or *O*-GlcNAcylation of the three gene products, OBP (*stricto sensu*, referred as OBP in the text below), Von Ebner's Gland Protein (VEG), and Salivary lipocalin (SAL). These two post-translational modifications (PTM) were unexpected for secreted proteins, as they usually occur in nucleus and cytosol of the cell to regulate the function of many proteins involved in most physiological processes [For review, see (10–12)]. Phosphorylation of secreted proteins is now largely documented. Some candidate kinases may modify the OBP sequence, such as the Golgi kinase FAM20C (13) or ectokinases (14). *O*-GlcNAc modification of proteins passing through the secretion pathway was only shown a decade ago (15), and since then the associated glycosyltransferase (EOGT) has been identified in Drosophila by (16). This enzyme is conserved throughout Evolution, including in the pig genome (9), but it is genetically unrelated to its cytosolic functional counterpart, the *O*-GlcNAc transferase OGT. To date, there is no evidence of a secreted enzyme that could act like the cytosolic *O*-GlcNAcase (OGA) to remove GlcNAc moieties.

In pig, phosphorylation drives binding specificity of recombinant OBP isoforms (17) that are not *O*-GlcNAcylated, at the contrary of native OBP, purified from nasal tissue (9). In order to precisely identify the role of both PTM in OBP binding abilities to pheromone components in pig, we purified OBP isoforms by HPLC and performed a structure-function relationship study. We identified phosphorylation and *O*-GlcNAcylation on 4 OBP isoforms by immunodetection with specific antibodies, along with careful controls. The binding properties of these 4 isoforms were monitored by fluorescence spectroscopy in the exact same conditions previously used for recombinant OBP isoforms (17). The PTM sites were mapped by CID-nano-LC-MS/MS and BEMAD (for phosphorylation) to link observed binding affinities to phosphorylation and *O*-GlcNAcylation patterns of native OBP isoforms. Comparison of binding affinities between recombinant (only phosphorylated) and native (also *O*-GlcNAcylated) isoforms indicates that *O*-GlcNAcylation increases their binding specificity to pheromone components. This is the first work that identifies such PTM sites in OBP using high-resolution-mass spectrometry, and demonstrates their involvement in the discrimination of odorant molecules by OBP isoforms.

## Materials and Methods

### Animals and Tissues

Animals (Large White *Sus scrofa*) were maintained at the Experimental Farm of INRA (UEPAO, Nouzilly, France). Nasal tissue was collected in a slaughterhouse, included in a surgical platform ISO9001-certified, which received European approval (N° E37-175-2) for the slaughter of domestic species like pigs. Respiratory mucosa (RM) was dissected from anesthetized animals (pentobarbital) immediately after death and stored in tubes at −80°C until protein extraction.

### Protein Purification and Characterization

Proteins were gently extracted from RM of pre-pubertal males of *S*. *scrofa* as described previously (7), in order to avoid intracellular protein release. Purification of OBP isoforms was achieved by high-resolution anion exchange chromatography on a ÄKTA purifier HPLC device (GE Healthcare). Proteins were separated on a PROPAC SAX10 column (Dionex, 4 mm × 250 mm), in 50 mM Tris/HCl, pH 7.5 (buffer A), by using an optimized gradient of 0–1 M NaCl (buffer B: 50 mM Tris/HCl, pH 7.5, 1 M NaCl): after 5 min in 100% buffer A, 50% of buffer B was reached in 30 min and maintained for 5 min, then 100% of buffer A was reached in 5 min, and then maintained for 10 min. Samples of 100 μl in buffer A were injected, and resulting fractions were collected, desalted with PD-10 desalting columns (GE Healthcare), and dried in a Speed-Vac (Eppendorf). Each fraction was re-purified on the same column but with a different NaCl gradient. Dried fractions were re-suspended into 100 μl buffer A, injected and purified with the following gradient: 7 min of 100% buffer A, 25% buffer B in 2 min, 35% buffer B in 10 min, 100% buffer B in 3 min, 7 min of 100% buffer B, then 100% A in 5 min. Resulting fractions were desalted with PD-10 desalting columns. Identical fractions coming from several injections were pooled to obtain homogenous aliquots (5 μg), dried in Speed-Vac and stored at −20°C until subsequent analyses. OBP isoforms were identified in HPLC fractions by western-blotting with anti-OBP antibodies (7). Their identity was confirmed by mass fingerprinting, followed by MALDI-TOF MS as already described (7). Protein concentration was determined by the Bradford method using recombinant porcine OBP (18) as standard (Micro BCA™ Protein Assay Kit, Pierce).

### One Dimensional and Two-Dimensional Electrophoresis

All chemicals and reagents were from Sigma-Aldrich, unless specified. For two-dimensional electrophoresis (2D-E), 5 μg of dried proteins were solubilized in 150 μl of the rehydration buffer (8 M Urea, 2 M Thiourea, 2% (w/v) CHAPS, 10 mM dithiothreitol (DTT), 1.2% (v/v) Immobilized pH Gradient (IPG) buffer (pH 4-7) (GE Healthcare) and bromophenol blue). After vigorous shaking, proteins were loaded onto a 7-cm IPG strip (pH 4–7, Bio-Rad) by overnight passive rehydration at room temperature. The first-dimensional isoelectric focusing (IEF) was carried out on a PROTEAN® i12™ IEF system (Bio-Rad) using the following program: 250 V for 30 min (rapid voltage ramping), 1,000 V for 1 h (gradual ramping), 5,000 V for 2 h (gradual ramping) and held at 5,000 V (rapid ramping voltage) until complete IEF (10,000 VH final), with a current limit at 50 μA/gel. Strips were then incubated twice for 15 min in the equilibration buffer (375 mM Tris-HCl pH 8.8, 6 M urea, 2% (w/v) SDS and 30% (v/v) glycerol) complemented with 1.5% (w/v) DTT, followed by 15 min in the equilibration buffer complemented with 2% (w/v) iodoacetamide. The second-dimension separation, as well as mono-dimensional electrophoresis, were performed using 16.8% SDS–PAGE in Mini PROTEAN® Tetra Cell (Bio-Rad) as already described (19).

### Staining and Western-Blot

After electrophoresis, gels were either stained with colloidal Coomassie blue R solution (12% trichloroacetic acid, 5% ethanolic solution of 0.035% Serva blue R 250) or transferred onto PVDF (ImmobilonP, Millipore) membranes. For immunodetection, membranes were blocked in 5% (w/v) non-fat dry milk in Tris-Buffered Saline with 0.05% (v/v) Tween 20 (TBS-T) for probing with polyclonal antibodies (home-made anti-OBP) and 3% BSA fraction V in TBS-T for probing with monoclonal anti-*O*-GlcNAc (RL2 and CTD110.6) antibodies or with anti-phosphoserine, -phosphothreonine, and -phosphotyrosine antibodies. Membranes were then incubated with antibodies in TBS-T 1 h at room temperature (RT) (RL2 (Thermofisher) 1:2,000; CTD 110.6 (Thermofisher), 1:5,000; anti-OBP, 1:30,000; anti-phosphoserine (P-Ser, 1:100), anti-phosphotyrosine (P-Tyr, 1:100), and anti-phosphothreonine (P-Thr, 1:500) (Invitrogen). After washes in TBS-T, membranes were incubated with the appropriate horseradish peroxidase-conjugated secondary antibody (anti-mouse IgG-HRP linked, 1:30,000, Thermofisher, for RL2; anti-mouse IgM-HRP linked, 1:30,000, Thermofisher, for CTD 110.6; anti-rabbit IgG-HRP linked, 1:30,000, Thermofisher for other primary antibodies) for 1 h at room temperature. After washes in TBS-T, blots were developed using enhanced chemiluminescence (ECL Plus Reagent, Hyperfilm™ MP, GE Healthcare). For GlcNAc competition assays, proteins were separated by SDS-PAGE, transferred onto PVDF membranes, which were blocked as above. Then, membranes were incubated 1 h (RT) with a pre-incubated mixture (1 h at RT) of CTD110.6 antibodies and 1 M GlcNAc (TCI, ref. A0092) in TBS-T-3% BSA. They were washed and processed as above until ECL detection.

### Mapping Post-translational Modification Sites in Native OBP

#### Mapping of Phosphorylation Sites by BEMAD

Phosphorylation sites of native OBP isoforms were mapped by mild Beta-Elimination, followed by Michael Addition of DTT (BEMAD) and then by MALDI-TOF MS analysis (20). Prior to mild β-elimination and Michael addition of DTT, isoforms were treated with β-*N*-acetylglucosaminidase (Sigma-Aldrich, 1 unit per 5 μg of protein) to remove GlcNAc groups. Proteins were not reduced and alkylated to avoid addition of DTT on cysteines. They were then digested with either trypsin (T) or chymotrypsin (CT) or both (T + CT) (Sigma-Aldrich). After a step of enrichment of DTT-modified peptides by using thiol columns [fully described in Nagnan-Le Meillour et al. (7)], peptides were eluted directly into the matrix (α-cyano-4-hydroxycinnamic acid) with 12.5, 25, and 50% acetonitrile (ACN). Eluates were not pooled in order to obtain higher percentages of peptide recovery in mass spectrometry. The theoretical masses of DTT-modified peptides were calculated from the OBP peptide map (GenBank accession number NP_998961) by using the Peptide Mass software at https://web.expasy.org/peptide_mass/.

#### Mapping of PTM Sites by CID Mass Spectrometry

##### Enrichment of O-GlcNAcylated proteins using wheat germ agglutinin columns

Samples were prepared for mass spectrometry by using WGA enrichment of GlcNAc-peptides. The WGA agarose beads (75 μL, Vector Laboratories, in Spin column-Screw caps, Pierce) were washed three times with wash buffer (100 mM Tris/HCl, pH 7.7) and spin down (12,000 rpm, MiniSpin Eppendorf), 5 min at 4°C. Total proteins (200 μg of total RM extract) were resuspended in 300 μL of wash buffer and incubated with WGA beads 2 h, at 4°C and 450 rpm (Thermomixer, Eppendorf). After 2 washes with wash buffer, GlcNAc-enriched proteins were eluted in Laemmli buffer, boiled and resolved on 1-D electrophoresis as described above. Bands were cut out and treated for trypsin digestion as described in Guiraudie et al. (19). Resulting peptides were desalted in reverse-phase C18 Spin columns (Thermofisher) and dried in Speed-Vac until use.

##### NanoLC-MS/MS analysis

NanoLC-MS/MS experiments were conducted on an Ultimate 3000-RSLC (Thermo Scientific, Massachusetts) using a C_18_ column (RSLC Polar Advantage II Acclaim, 2.2 μm, 120 Å, 2.1 Å~100 mm, Thermo Scientific, Massachusetts) at a flow rate of 300 μL/min using a mobile phase gradient with A: H_2_O + formic acid (FA) 0.1% and B: acetonitrile (ACN) + FA 0.08%. The gradient increased from 2% B to 25% B in 60 min and then to 60% B in 65 min. One microliter of sample was injected and the separated peptides were analyzed by a Q-TOF mass spectrometer (quadrupole-time of flight instrument, Maxis II ETD, Bruker Daltonics, France) interfaced with an ESI (electrospray ionization) source. The Nano spray source was operated at 1,600 V with a nano-booster system and the MS spectra were recorded at 2 Hz between *m*/*z* = 150 to 2,200. MS/MS spectra were acquired for precursor ions between *m/z* = 400 to 2,200 with charge states from +2 to +5. Fragmentation rate varied between 1 to 4 Hz depending on the precursor ion intensities. Total cycle time was fixed at 3 sec. Active exclusion time was set to 0.5 min to favor the MS/MS of low intensity ions.

##### Data analysis

The LC-MS/MS analyses were processed and converted into ^*^.mgf files using Data Analysis software (version 4.3.110, Brüker Daltonics). Database search was carried out using in-house Mascot software (version 2.4.0, MatrixScience.com, London, UK) with following parameters: MS tolerance = 10 ppm, MS/MS tolerance = 0.05 Da, carbamidomethylation of cysteine as fixed modification, oxidation (methionine), pyrrolidone carboxylic acid (Q), phosphorylation (S, T, Y), HexNAcylation (S, T), and Q/N deamidation as variable modifications. Up to two trypsin missed cleavages were allowed. A custom database containing *S. scrofa* OBP isoforms and splicing variants (Supplementary Data 1) was used. All the ions in the peak list generated using Data Analysis software were checked manually to construct fragment ion tables and to perform annotation of the spectra with the aid of a Protein Prospector (v 5.22.1 at prospector.ucsf.edu/). The mass spectrometry proteomics data has been deposited to the ProteomeXchange Consortium via the PRIDE (21) partner repository with the dataset identifier PXD011371 and 10.6019/PXD011371.

### Fluorescence Binding Assay

Androstenone (3-keto-5α, 16-androstene), androstenol (3α-hydroxy-5α-androst-16-ene), testosterone (17β-hydroxy-3-oxo-4-androstene), palmitic acid (hexadecanoic acid), myristic acid (tetradecanoic acid), and AMA (1-aminoanthracene) were purchased from Sigma-Aldrich. UV-visible spectra were recorded on a Cary-100 double beam spectrometer (Varian) with a cell of 0.5 cm path length. The protein concentration was calculated by UV-visible spectroscopy, by using the molar extinction coefficient of 11,740 M^−1^cm^−1^ for OBP (calculated by the software “ProtParam tool” at www.expasy.org) at the maximum wavelength of absorption (280 nm). Steady state fluorescence measurements were performed on a Fluoromax-3 (Jobin-Yvon) spectrofluorimeter using a micro quartz cell (0.5 cm light-path) with a reflecting window. 1-Aminoantracene (AMA) was used as a fluorescent probe. The experimental conditions and data treatment have been described previously (17). The dissociation constant (K_d_-AMA) of the OBP-AMA complex was calculated from the binding curve by fitting the experimental data with the use of the computer program Origin 7.5 (OriginLab Corporation). The affinities of the five ligands were measured in a competitive binding assay with AMA as the fluorescent probe. AMA was dissolved in 100% ethanol to yield a 1 mM stock solution. For protein titration, aliquots of AMA were successively added to the protein at 1 or 2.5 μM in 50 mM Tris buffer (pH 7.8) and emission spectra were acquired after a 15 min equilibration period. Competitive binding experiments were then carried out with different protein and ligand concentrations. The spectra were recorded 15 min after the ligand addition, and competition was monitored by following the decrease in intensity of fluorescence associated with the band of AMA bound to the protein. The apparent K_d_ values of the ligand-protein complexes were calculated as described in Brimau et al. (17).

## Results

### Purification and Identification of Native OBP Isoforms

**Figure 1:**
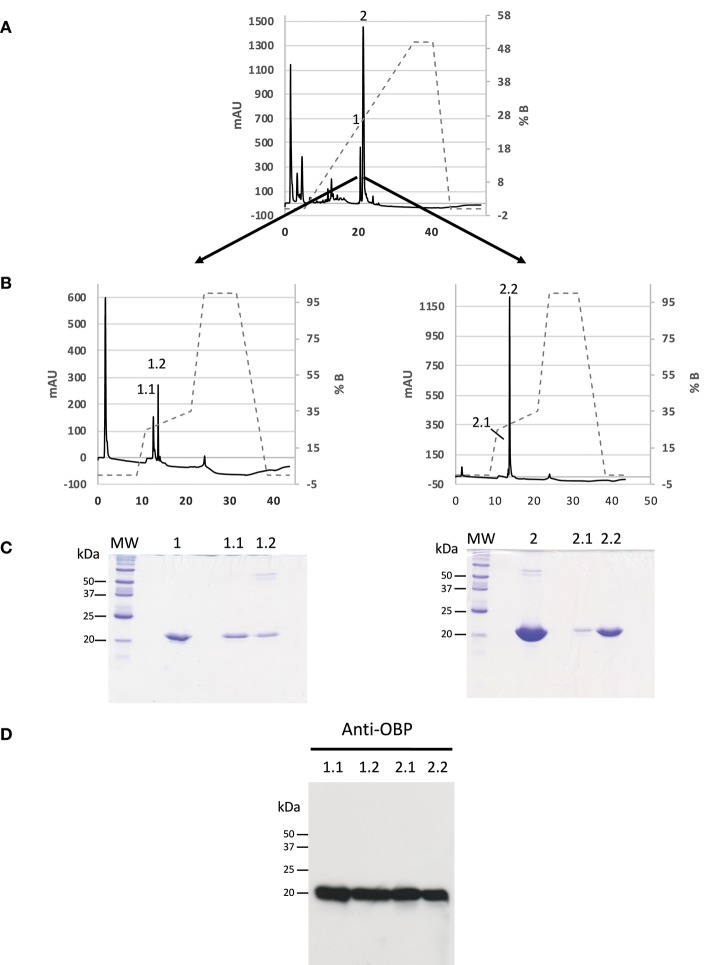
Purification of OBP isoforms from *S. scrofa* by anion-exchange HPLC. Dotted lines represent the gradient of NaCl. **(A)** Chromatogram of the first step of purification from a total RM extract of pre-pubertal male indicating the fractions 1 and 2 containing OBP. **(B)** Proteins of fractions 1 and 2 were separated in a second round of purification leading to two peaks in each fraction. Absorbance was monitored at 215 nm. **(C)** Coomassie blue staining of 16.8% SDS-PAGE of fractions collected in the two successive rounds of purification (molecular weight markers Precision Plus Protein All Blue, BioRad). **(D)** Western-blot with anti-OBP antibodies (5 μg of each fraction, 1:20,000 dilution, ECL Plus detection, 15 s exposure).

Since phosphorylation could confer different global charges to isoforms, we used strong anion exchange chromatography with two successive steps of NaCl gradient to purify OBP isoforms from the crude RM extract from pre-pubertal males. The fractions resulting from the first round of purification (Figure 1A) were desalted, dried under vacuum (Speed-Vac) and resuspended in sample buffer to be analyzed by 1D SDS-PAGE (Figure 1C). Only fractions 1 and 2 were positive after labeling with anti-OBP antibodies (data not shown) and were submitted to the second round of purification, by using an optimized gradient to obtain a better separation of the two peaks containing OBP isoforms (Figure 1B). From each peak of the first purification 1 and 2, two fractions were separated (Figure 1C), eluted at 0.266 M (fraction 1.1), 0.276 M (fraction 1.2), 0.277 M (fraction 2.1), and 0.280 M (fraction 2.2) of NaCl. The four fractions were immunoreactive to anti-OBP antibodies (Figure 1D). Each fraction was individually analyzed by two-dimensional electrophoresis (Supplementary Figure 1). For OBP identification, mass fingerprinting was performed on the 2D-E gel spot of each fraction (Supplementary Table 1, spectra in Supplementary Figures 2–5), giving 75.1% of peptide recovery for fraction 1.1 (referred to as OBP-native-iso1), 61.8% for fraction 1.2 (OBP-native-iso2), 90.4% for fraction 2.1 (OBP-native-iso3), and 100% for fraction 2.2 (OBP-native-iso4). In the theoretical map of trypsin digestion, two peptides allow discrimination between X1 and X2 variants: peptide 73–87 of *m/z* 1686.7354 specific to the X1 (only found in fraction 2.2), and peptide 73–85 of *m/z* 1498.6227 specific to X2. Unfortunately, the *m/z* of the later is closer to the one of peptide 16–28 (1498.7424), which is common to the two variants (in red in Supplementary Table 1). The peptide of expected *m/z* 2439.1820, indicating an additional Lys in C-terminal end (C-term: PAK, 138–158 in X1, and 136–155 in X2), was retrieved only in OBP-native-iso3 (*m/z* 2439.8971), together with the peptide of m/z 2296.6929, typical of PA C-term.

### Immunodetection of Post-translational Modifications on Native OBP Isoforms

**Figure 2:**
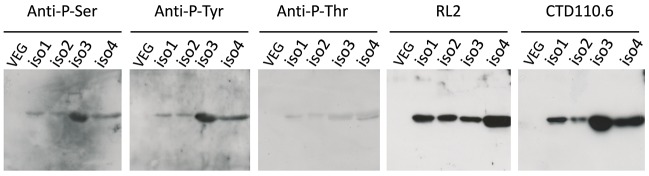
Immunodetection of phosphorylation and *O*-GlcNAcylation of OBP isoforms from *S*.*scrofa*. Each well contains a normalized quantity (5 μg) of the four HPLC fractions. HPLC-purified short-size VEG was used as negative control. **Anti-P-Ser**: anti-phosphoserine antibodies (1:500 dilution); **Anti-P-Tyr**: anti-phosphotyrosine antibodies (1:2,000); **Anti-P-Thr**: anti-phosphothreonine antibodies (1:500); **RL2** (1:2,000), **CTD110.6** (1:4,000). Secondary antibodies for anti-phosphorylation antibodies at 1:40,000 dilution (rabbit IgG-HRP linked whole antibodies), ECL Plus detection (11 min exposure). Secondary antibodies for *O*-GlcNAc detection: goat anti-mouse IgG-HRP linked (1:30,000) for RL2, and rabbit anti-mouse IgM-HRP linked (1:30,000) for CTD110.6, ECL Plus detection (30 s exposure).

Aliquots containing the same quantity of each fraction (5 μg) were analyzed by western-blot with specific antibodies raised against the three types of phosphorylation (anti-P-Ser, -P-Tyr, and -P-Thr) and against *O*-GlcNAcylation, RL2 and CTD110.6. The four OBP fractions were immunoreactive to the five antibodies (Figure 2). To test the specificity of antibodies, a short-size VEG (9) that is not phosphorylated nor *O*-GlcNAcylated (Figure 2) was used as a negative control and was not reactive with any antibodies. The specificity of the RL2 and CTD110.6 antibodies was further confirmed by western-blotting on two samples of RM total extract, one untreated and the other treated with β*-N*-acetylglucosaminidase. RL2 antibodies did not label the treated samples, indicating that the enzyme removed the GlcNAc groups, which assessed the β-linkage of GlcNAc moieties (Supplementary Figure 6A). Finally, a competition assay with free GlcNAc was performed to check antibody specificity. In these conditions, no signal was detected (Supplementary Figure 6B).

### Mapping of Phosphorylation and *O*-GlcNAcylation Sites

#### Analysis of OBP Phosphorylation by BEMAD

The phosphorylation sites of native OBP isoforms were mapped by using the BEMAD method in the same conditions as previously used for recombinant OBP (17), which is not *O*-GlcNAcylated, unlike native OBP isoforms (Supplementary Figure 7). DTT addition confers a tag of defined molecular mass to peptides bearing phosphate groups: 136.2 Da for one DTT, and 272.4 Da for 2 DTT. We used both T and CT enzymes to obtain short peptides allowing a more accurate localization of phosphate groups. The measured masses of peptides obtained after BEMAD treatment were compared to the theoretical list of potential DTT-modified peptides calculated from the OBP protein sequence (GenBank accession number NP_998961). The BEMAD results are given in Table 1 and the corresponding spectra are provided in Supplementary Figures 8–11. This method allowed potential localization of phosphorylation on Ser13, Ser41, Ser49, Ser57, and Thr 122 in OBP-native–iso1, Ser57, and Thr122 in –iso2, Ser23, Ser24, and Ser41 in –iso3, and Ser67 and Thr71 in –iso4. Since the mild beta-elimination does not open the aromatic cycle of tyrosine, PTM that bear this amino acid cannot therefore be detected by this method. However, Tyr residues of OBP isoforms could also be phosphorylated and only localized by high-resolution mass spectrometry. It is widely reported in the literature that the BEMAD method allows localization of phosphorylation with high confidence (22). Indeed, the action of β*-N*-acetylglucosaminidase allows a total elimination of GlcNAc moieties from the primary structure (Supplementary Figure 6A). Conversely, we have observed that alkaline phosphatase did not totally remove the phosphate groups, as phosphorylation is still detectable by specific antibodies after action of the enzyme (7). The BEMAD performed with alkaline phosphatase on OBP isoforms did not obtain confident results in *O*-GlcNAc site localization (data not shown). We thus performed mass spectrometry analyses to get more information on OBP isoforms PTM.

**Table 1:** Beta-elimination followed by Michael addition of dithiothreitol (BEMAD, DTT) performed on OBP isoforms to identify phosphorylation sites.

**Calculated mass (Da)**			**Measured mass (M + H)^**+**^**	**Peptide**	**Peptide sequence**	**Modification site**
**No DTT**	**1 DTT**	**2 DTT**				
**OBP-NATIVE-ISO1**						
1711.7809	1847.9808		1847.4896 (T, 50%)	1–15	QEPQPEQDPFELS(*)GK	S13
1140.5354	1276.7354		1276.6440 (T+CT, 50%)	39–47	MRS(*)IEFDDK	S41
1357.6634	1493.8634		1493.7390 (T+CT, 50%)	45–55	DDKES(*)KVLNF	S49
1155.4986	1291.6986		1291.3486 (T, 25%)	57–66	S(*)KENGICEEF	S57
1262.5746	1398.7746		1398.6817 (T, 25%)	121–131	GT(*)DIEDQDLEK	T122
**OBP-NATIVE-ISO-2**						
1140.5354	1276.7354		1276.5677 (T+CT, 50%)	57–66	S(*)KENGICEEF	S57
1262.5746	1398.7746		1398.8314 (T+CT, 50%)	121–131	GT(*)DIEDQDLEK	T122
**OBP-NATIVE-ISO3**						
1950.9807		2223.3807	2223.5032 (CT, 50%)	21–38	IGS(*)S(*)DLEKIGENAPFQVF	S23–S24
1140.5354	1276.7354		1276.8341 (T+CT, 25%)	39–47	MRS(*)IEFDDK	S41
**OBP-NATIVE-ISO-4**						
1539.7359		1812.1359	1812.1110 (T, 25%)	59–72	ENGICEEFS(*)LIGT(*)K	S67–T71

Peptides derived from MALDI-TOF MS analysis after enrichment by thiol chromatography (Trypsin T, Chymotrypsin CT, both T+CT) and elution at different percentages of acetonitrile (12.5, 25, and 50%). *Denotes mass addition of 136.2 Da at a S or T residue, indicating modification by dithiothreitol (DTT) and potential phosphorylation.

#### Analysis of OBP PTM by High-Resolution Mass Spectrometry

Although we obtained 100% sequence coverage for OBPs from Mascot search, which was expected from HPLC purification and peptide mapping data, only few peptide matches could be identified bearing a PTMs. In fact, <10 out of around 10,000 spectra from total RM OBP and HPLC fractions gave relevant data on PTM presence with 4,987 unassigned peptides. Only ions differing by a Δ *m/z* < 0.01 from theoretical masses given by Protein Prospector were taken into consideration for annotation of spectra and ion tables (Supplementary Tables 2–4). Most of the data that confidently allows PTM site localization came from the analysis of the total OBP sample, which was enriched in GlcNAc-proteins on WGA column, at the contrary of the HPLC fractions. In preliminary assays, no result was obtained from an individual HPLC fraction, but peak 1 and peak 2 samples resulting from the first purification round gave information about phosphorylation sites (see below).

**Figure 3:**
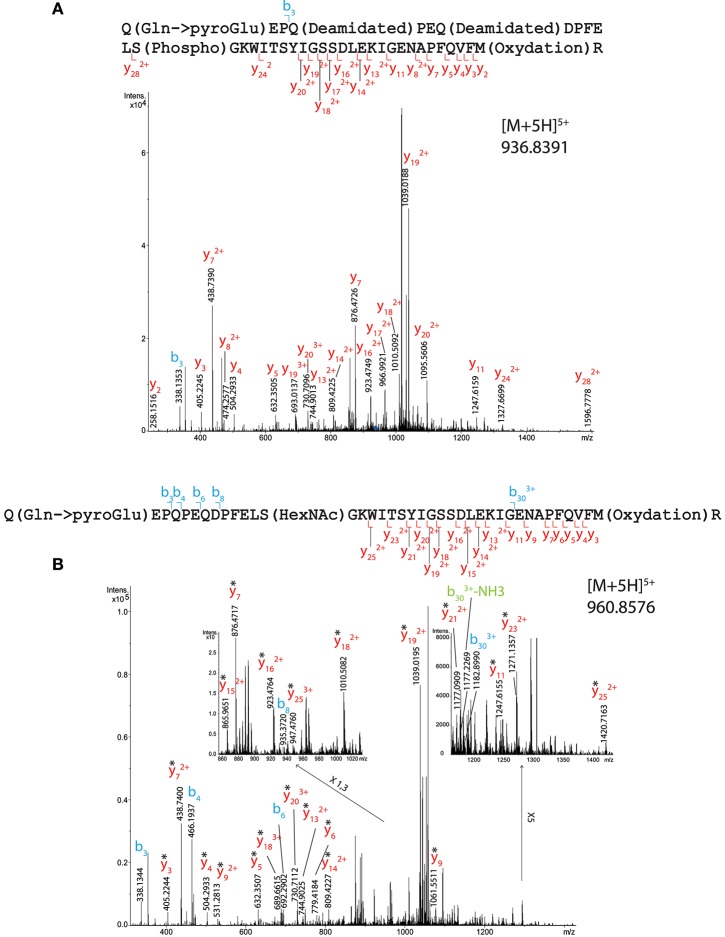
MS/MS CID fragmentation spectra of tryptic peptide 1–40 from total RM extract identifies **(A)** phosphate modification site S13 or **(B)** HexNAc modification site S13. *Denotes loss of SOCH_4_ (oxidation) on y-series.

Serine 13 was assigned to bear either a phosphate or a GlcNAc group (Figure 3) in tryptic peptide 1–40 (QEPQPEQDPFELSGKWITSYIGSSDLEKIGENAPFQVFMR, Mascot scores of 28 and 46, respectively) coming from a total OBP sample. The phosphorylated peptide was detected as [M+5H]^5+^ species at *m/z* 936.8391. Its CID spectrum is shown on Figure 3A and the list of fragment ions is provided in Supplementary Table 2). The phosphate group could unambiguously be located at S13 from y24^2+^ and y17^2+^ fragment ions. The HexNAcylated peptide was detected as [M+5H] ^5+^ species at *m/z* 960.8576. Its CID spectrum is shown on Figure 3B with the list of fragment ions provided as Supplementary Table 3. This peptide also carried a methionine oxidation at M39, resulting in - SOCH_4_ neutral losses on the y-series. The CID spectrum revealed a b30^3+^ ion carrying and HexNAc moiety, while none of the y ions detected (up to y_25_) showed HexNAcylation. This suggests that the GlcNAc modification is localized at S13, although the lability of GlcNAc moiety upon CID cannot permit to rule out additional modification sites in region 1–40.

**Figure 4:**
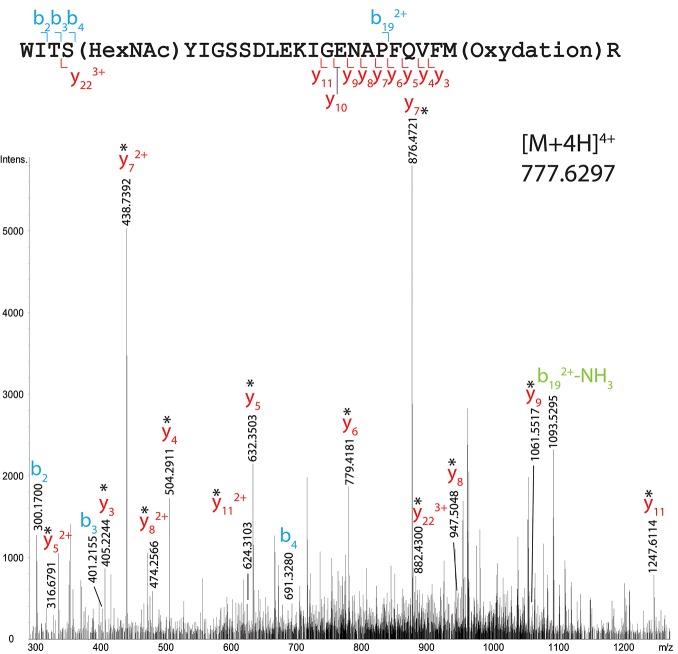
MS/MS CID fragmentation spectrum of tryptic peptide 16–40 from total RM extract identifies HexNAc modification site S19. *Denotes loss of SOCH_4_ (oxidation) on y-series.

Indeed, another site of HexNAcylation was revealed at S19 in peptide 16–40 from total OBP sample (WITSYIGSSDLEKIGENAPFQVFMR, Mascot score 19). The species [M+4H]^4+^ at *m/z* 777.6297 corresponds to a mass shift of 203.0732 Da compared to the unmodified peptide, matching with the theoretical mass of one HexNAc moiety (203.0794 Da). The CID spectrum is shown on Figure 4 and the list of fragment ions is provided as Supplementary Table 4. The fragment ions b_4_ and y_22_^3+^ ions permitted to unambiguously localize the HexNAc at S19.

**Figure 5:**
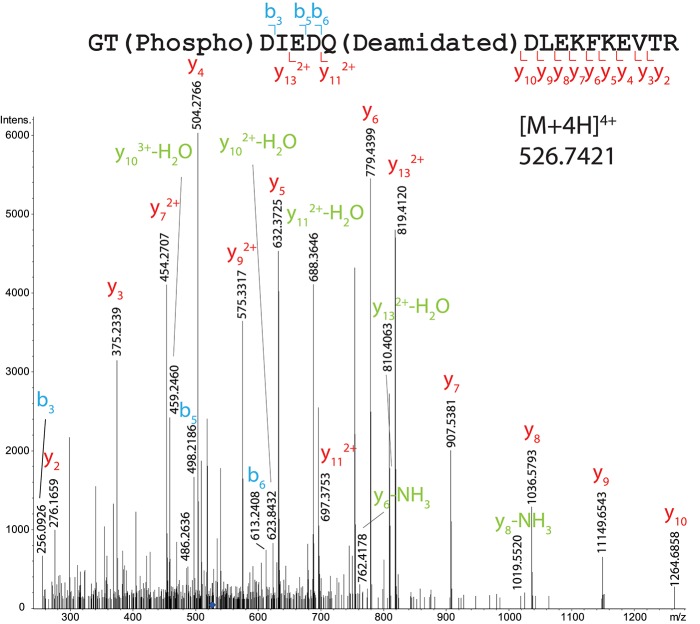
MS/MS CID fragmentation spectrum of tryptic peptide 121–138 from total RM extract identifies phosphate modification site T122.

One phosphorylation site has been localized on T122, on the peptide 121–138 (GTDIEDQDLEKFKEVTR) both in total OBP (Figure 5 and Supplementary Table 5) and HPLC fraction (peak 2 of the first round of purification containing OBP-native-iso3 and -iso4, Supplementary Figure 12). In total OBP sample data (Mascot score 36), the mass difference between experimental mass of [M+4H]^4+^ = 526.7421 and the one calculated for naked peptide is *m/z* 19.9912, giving an addition of *m/z* 79.9648 close to the theoretical mass of the phosphate group (79.9668, mass Δ of 0.002). Ions b3, b4, and b6 indicate that T122 bears the phosphate moiety but not T137. Besides this site localization, other amino acids were identified as potentially modified by phosphorylation. The mass of the peptide 112–133 of sequence TIMTTGLLGKGTDIEDQDLEKFK (HPLC peak 1 containing OBP-native-iso1 and -iso2) indicates the presence of two phosphate groups. Indeed, parent ion [M+5H]^5+^ with *m/z* = 523.2381 corresponds to the mass of the unmodified peptide plus two phosphorylation sites, but we could only confirm the presence of one phosphate by manual analysis of the MS/MS spectrum (Supplementary Figure 13). T112 or T115 could be phosphorylated as b5 ion mass corresponds to only one phosphate. In this peptide, T122 is not phosphorylated, which is assessed by ions y2 to y13^2+^, and ions b12^2+^ and b13^2+^, the masses of which correspond to the naked peptide.

### Monitoring of Ligand Binding by Fluorescence Spectroscopy

**Figure 6:**
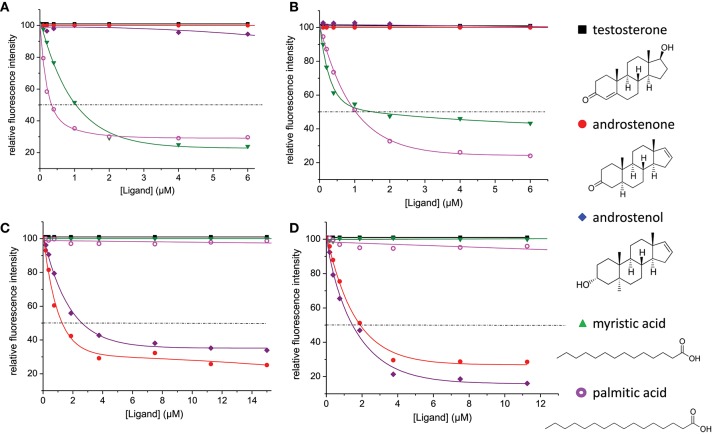
Competitive binding assays of five selected ligands of biological relevance to native OBP isoforms from S. scrofa. **(A)** OBP-native-iso1; **(B)** OBP-native-iso2; **(C)** OBP-native-iso3; and **(D)** OBP-native-iso4. Proteins were used at 2.5 μM **(A,B,D)** or 1 μM **(C)** concentration and were saturated with 3.75 μM **(A,B,D)** or 2 μM **(C)** of the fluorescent probe 1-AMA. Competitors were added at increasing concentration from 0 to 6 μM **(A,B)** or to 15 μM **(C,D)**.

We measured the affinity of the four native OBP isoforms for the five selected ligands under the same experimental conditions that were used previously for recombinant OBP isoforms (17). The four isoforms were first titrated with the fluorescent probe, AMA. OBP-native-iso3 was collected in smaller quantities than the 3 other isoforms and was used at a 1 μM concentration instead of 2.5 μM and saturated with 2 μM AMA, instead of 3.75 μM AMA. The four isoforms displayed a comparable affinity for AMA: the estimated K_d−AMA_ were 0.37 ± 0.02, 0.23 ± 0.02, 0.84 ± 0.08, and 0.50 ± 0.02 μM for OBP-native-iso1, -iso2, -iso3, and -iso4, respectively. Ligands were then added, displacement curves are reported in Figure 6 and binding constants calculated are listed in Table 2. None of the four native isoforms bound testosterone. Two classes of isoforms, -iso1 and -iso2, bound the two fatty acids and not the steroids. The opposite binding specificity was observed for -iso-3 and –iso-4 (Table 2).

**Table 2:** Values of [IC50] and calculated dissociation constants (K_d_) relative to the binding of ligands to native OBP isoforms.

**Ligands**	**OBP-native-iso1**		**OBP-native-iso2**		**OBP-native-iso3**		**OBP-native-iso4**	
	**IC_**50**_**	**K_**d**_**	**IC_**50**_**	**K_**d**_**	**IC_**50**_**	**K_**d**_**	**IC_**50**_**	**K_**d**_**
Testosterone	–	–	–	–	–	–	–	–
Androstenone	–	–	–	–	1.28	0.23	1.89	0.22
Androstenol	–	–	–	–	2.59	0.49	1.43	0.16
Palmitic acid	1.03	0.16	1.51	0.16	–	–	–	–
Myristic acid	0.33	0.05	1.03	0.11	–	–	–	–

*IC_50_ and K_d_ are expressed in μM*.

## Discussion

Competition assays between fluorescent probes and odorant ligands or pheromone components are largely used in olfaction for testing the binding properties and measuring binding affinities of olfactory binding proteins from both insects and mammals. Most of the works were performed on recombinant OBPs produced in bacteria (*Escherichia coli*) or in yeast (*P. pastoris*), the former being unable to perform mammalian PTMs, and the later lacking of a coding sequence for OGT and EOGT in its genome (Ensembl database search). Since we were the first to evidence the presence of phosphorylation (7) and *O*-GlcNAcylation on a mammalian OBP (8, 9), the purpose of this work was to compare the binding abilities of Pichia-recombinant OBP isoforms with those of native OBP isoforms purified from nasal mucus, and to link their binding affinities to their PTM pattern. Functional assays were performed in the exact same conditions for recombinant (17) and native isoforms, with specific ligands of the pig species chemical communication: the palmitic and myristic fatty acids are part of the maternal appeasing pheromone (19, 23), and androstenol and androstenone compose the sex pheromone of the boar saliva (24).

### Native OBP Isoforms Display Different Binding Affinities to Specific Ligands

**Figure 7:**
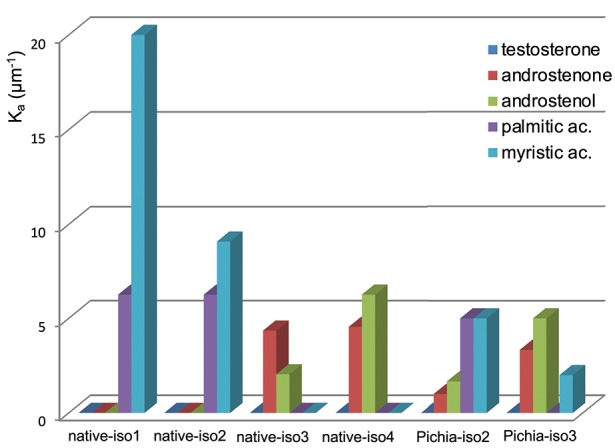
Calculated affinity constants (Ka) for the different isoforms of *S. scrofa* OBP-native (this work) and OBP-Pichia [from Brimau et al. (17)] toward the five tested ligands.

For direct comparison and clarity purposes, the affinities of OBP isoforms [-native from this work and -Pichia from Brimau et al. (17)] toward each different ligand were expressed in K_a_ values of the complexes obtained (Figure 7). As expected, none of the four isoforms bound testosterone, since testosterone is the specific ligand of the VEG, another protein of the nasal mucus of *S. scrofa* (8), and was used as negative control. This result demonstrates that differences between binding affinities reflect OBP isoform specificity and do not result from experimental artifacts since the three steroids have similar chemical structures, which are discriminated by OBP isoforms. Indeed, OBP-native-iso1 and -iso2 only bind both fatty acids, with a higher affinity for myristic acid than for palmitic acid. The important binding ability (K_d_ = 50 nM) of OBP-native-iso1 for myristic acid reflects the highest affinity ever reported by fluorescence spectroscopy for an OBP, regardless of its origin (species, native or recombinant) [e. g., (25–28)]. In contrast, OBP-native-iso3 and -iso4 have no affinity for fatty acids, but they bind androstenone and androstenol. Although the affinities of these two isoforms for androstenone are comparable, the K_a_ value for androstenol is three times greater for OBP-native-iso4 than for OBP-native-iso3. Compared to the OBP-Pichia, the native OBP isoforms appear to have significantly more specific binding power. OBP-Pichia-iso2 more readily bound to fatty acids, but displacement of the fluorescence probe was still observed with the addition of steroids. Conversely, OBP-Pichia-iso3 preferentially bound to the steroids, but also to a lesser extent to myristic acid, but was not able to bind palmitic acid. The lower K_a_ values and low selectivity of the OBP-Pichia-iso2 and -iso3 compared to the OBP-native isoforms cannot be explained by primary sequence differences or by the crystallographic structure previously published (29–31), but more likely can be explained by different patterns of post-translational modifications.

### CID-MS/MS Allowed Identification of Phosphorylation and *O*-GlcNAcylation Sites on OBP Sequence

**Figure 8:**
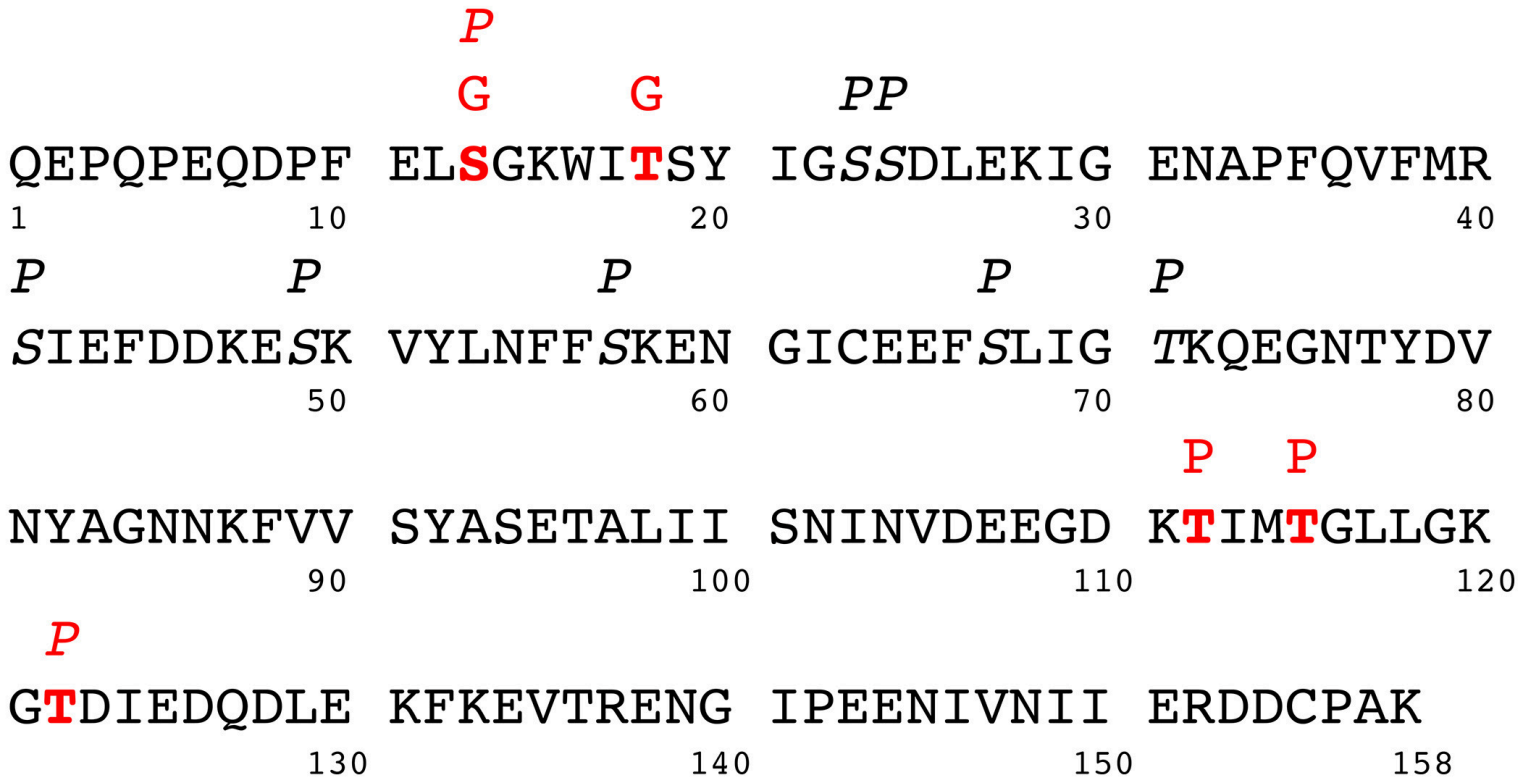
Amino acid sequence of porcine OBP (GI/47523248 - OBP X1j) with PTM sites: P or G above an amino acid symbol indicate a phosphorylation or an HexNAcylation site. PTM sites identified by CID-MS/MS are highlighted in bold and red—phosphorylation: S13, T112 or T115, T122—HexNAcylation: S13, T19. Phosphorylation sites identified by BEMAD are in italic type: S13, S23, S24, S41, S49, S57, S67, T71, T122.

Although pig secretes a large quantity of OBP, HPLC purification in one side and enrichment in glycoproteins (WGA columns) or phosphopeptides (thiol columns in BEMAD protocol) in the other side resulted in considerable loss of relevant information on PTMs in mass spectrometry analyses. MR extract from a single animal provides just enough material to perform HPLC purification followed by western-blots, BEMAD and mass spectrometry analyses. Given that preliminary assays in glycoprotein enrichment after HPLC purification indicated a near total loss of sample (no PTM signal in western-blot was detected), this enrichment protocol was only performed on total MR extract, so on a mixture of OBP isoforms. In addition, it is known that in MS, the presence of unmodified peptide ions drastically suppresses the ionization of HexNAc-peptides, much more so than for phospho-peptides (32). The WGA enrichment protocol was performed on a complex pool of total OBP isoforms containing glycosylated isoforms that were retained on the column. Once eluted, they were digested by trypsin, resulting in a mixture still containing unmodified and modified peptides with a ratio favorable to unmodified peptides, as observed in mass spectrometry. For nuclear and cytoplasmic proteins, *O*-GlcNAcylation has a low stoichiometry at each site, for instance, crystallin, a protein highly *O*-GlcNAcylated, exhibits an *O*-GlcNAc stoichiometry as low as 2% (33). Our analysis suggests that *O*-GlcNAcylation of OBP isoforms would fall within a similar range, leading to a difficult identification of HexNAc-sites, independently of the ionization source. Consequently, CID-MS/MS on HPLC fractions gave scarce information on fractions PTM patterns. Nevertheless, several phospho-sites and HexNAc-sites were confidently identified for the first time on a mammalian OBP. Ascertained and potential modification sites obtained with BEMAD and Mascot analysis of CID MS/MS are summarized in Figure 8. Enrichment of GlcNAc peptides could be achieved through using click-chemistry (34, 35) coupled to Electron Transfer Dissociation-MS/MS [ETD-MS/MS, for a review see Ma and Hart (32)] or negative ion CID-MS/MS (36), to improve the coverage of all OBP PTM sites. Nevertheless, glycopeptide enrichment is required to obtain confident data with ETD-MS/MS, which is far less sensitive than the CID mode.

Interestingly, the sites of phosphorylation localized in the total native OBP (7) were all retrieved and dispatched among the 4 isoforms. MS/MS analysis identified two phospho-sites, and confirmed their localization at S13 and T122. Phosphorylation of secreted proteins is nowadays well documented and some candidate kinases could modify the OBP sequence. FAM20C, a Golgi kinase, is responsible for phosphorylating the majority of secreted phosphoproteins at the S-x-E motif and could thus modify OBP at S41 (S-I-E), and S57 (S-K-E). But extensive analysis of secreted phosphoproteins suggests that FAM20C could target substrates lacking this canonical recognition site (13). In this work, no phosphorylation was identified on tyrosine residues by CID-MS, despite the labeling of the four fractions by anti-phosphotyrosine antibodies, particularly strong for OBP-native-iso3 (the less abundant OBP isoform) (Figure 2). However, in previous work (37), we have identified phosphosites, which were not all retrieved in this study, particularly at Y78 or Y82. The use of different mass spectrometry conditions (HCD-Orbitrap) and different animals could explain this discrepancy. Phosphorylation on OBP could be done by the vertebrate lonesome kinase (VLK), a secreted kinase that modifies extracellular proteins on Y residues (14). The absence of identified phosphoY-peptides in our MS data is most likely due to their faint quantity compared to phosphoS/T-peptides (22). In vertebrate cells, the phosphoS:phosphoT:phosphoY ratio is 1800:200:1. Most cytosolic S/T kinases recognize a highly basic consensus sequence, which is the case for five sites predicted by BEMAD and/or CID-MS (R-S41-I, E-S49-K, F-S57-K, G-T71-K, K-T112-I). Despite extensive research that has been reported in the literature and the data that we provide here, the kinase (s) responsible for OBP phosphorylation still remain (s) to be identified.

Two sites of HexNAcylation were unambiguously localized at S13 and S19 of OBP sequence. Due to the novelty of our findings, we were extremely cautious in data acquisition and interpretation. The β-linkage of the HexNAc moiety was demonstrated by both western-blots with specific CTD110.6 and RL2 antibodies and by β-N-acetylglucosaminidase treatment. The competition assay with free GlcNAc confirmed the specificity of CTD110.6 antibodies against *O*-GlcNAc modified OBP. Indeed, it has been reported that CTD110.6 could recognize *O*-GlcNAcylated cell surface proteins, such as Notch1, but also terminal β-GlcNAc of complex N- or O-glycans (38), that could not be the case for OBP. The protein has no consensus site for either N- or O-complex glycosylation, and treatments with appropriate enzymes have confirmed their absence in the past (39). *O*-GlcNAcylation has been thought to be restricted to nuclear and cytoplasmic proteins, and in a lesser extent, mitochondrial ones, until the report of *O*-GlcNAcylation of proteins processed in the secretion pathway (15). The enzyme, named EGF-domain specific *O*-GlcNAc transferase (EOGT) transfers a GlcNAc moiety to proteins with an epidermal growth factor (EGF) repeat on their extracellular domain, such as Notch1 or Dumpy (15, 40). EOGT is ER-resident and so, can theoretically modify any protein that passes through the secretion pathway, not only the membrane attached proteins. Very few proteins secreted in the extracellular medium were reported to be *O*-GlcNAc modified. But findings from Alfaro et al. (41) suggest that EOGT could target additional substrates because some *O*-GlcNAc sites were found on proteins lacking the EGF domain and the consensus motif C_5_XXGXS/TGXXC_6_; porcine OBP also lacks both of them. Among 274 *O*-GlcNAc proteins identified in the *O*-GlcNAcome of rodent brain tissue, only 5 membrane proteins and one secreted cytokine were *O*-GlcNAcylated (41). Therefore, contrary to the assessment that EOGT modifies only secreted and membrane proteins that contain one or more epidermal growth factor-like repeats with a specific consensus sequence, we demonstrated that secreted OBP, without such features in its sequence, is *O*-GlcNAc modified and could be a substrate for the porcine EOGT, already characterized in respiratory mucosa tissues (9).

### *O*-GlcNAcylation Increases Both Specificity and Affinity of OBP Isoforms

Recombinant OBP-Pichia isoforms that are phosphorylated bind steroids and fatty acids with slight differences. Conversely, native isoforms, that are phosphorylated but also *O*-GlcNAcylated, show radically different binding affinities for the same compounds, strongly suggesting that *O*-GlcNAcylation increases the binding specificity of OBP isoforms. Moreover, one of the OBP isoforms, OBP-native-iso1, labeled by CTD110.6 antibodies, displayed the highest affinity ever reported for an OBP. Phosphorylation and *O*-GlcNAcylation patterns seem to determine the binding specificity of isoforms, and cause a subset of OBPs to bind specific chemical classes of ligands with affinities in the nanomolar range. The identification of at least two *O*-GlcNAc sites, S13 and S19, interestingly localized on the flexible N-terminus part of the protein (30), will lead to side-directed mutagenesis followed by binding assays to better understand how this little sugar can modify OBP binding properties. Hence, phosphorylation and *O*-GlcNAcylation act together to generate OBP isoforms that are able to perform a first coding of odorant and pheromonal molecules. This suggests that a specific OBP-ligand complex would specifically interact with a given olfactory receptor, highlighting a new olfactory transduction mechanism. As G-protein coupled olfactory receptors (e. g. OR 585; accession number IPI00127945) have been identified to be *O*-GlcNAcylated (35), this exquisite coding might be mediated by sugar-sugar interactions. These findings extend the role of *O*-GlcNAc in regulating the function of many proteins involved in most, if not all, metabolic processes, including extracellular signaling in olfaction.

## Data Availability Statement

The mass spectrometry proteomics data have been deposited to the ProteomeXChange consortium via the PRIDE partner repository with the dataset identifier PXD011371 and 10.6019/PXD011371. The raw data (western-blot) supporting the conclusions of this manuscript will be made available by the authors, without undue reservation, to any qualified researcher.

## Author Contributions

PNLM, CLD, SZ, and JPC contributed to conception and design of the study. CLD performed peptide mapping and MALDI-TOF experiments. PNLM and AJ performed HPLC purification and western-blots. JPC conducted and analyzed fluorescence spectroscopy data. AM and SZ acquired and analyzed nano-LC-MS/MS data. PNLM and AJ analyzed nano-LC-MS/MS data. All authors wrote sections of the manuscript and contributed to manuscript revision, read, and approved the submitted version.

### Conflict of Interest Statement

The authors declare that the research was conducted in the absence of any commercial or financial relationships that could be construed as a potential conflict of interest.
