# Supplementary material for: Binding Specificity of Native Odorant-Binding Protein Isoforms Is Driven by Phosphorylation and O-N-Acetylglucosaminylation in the Pig Sus scrofa
Source: Front Endocrinol (Lausanne). 2019 Jan 25;9:816. doi: 10.3389/fendo.2018.00816 (PMC6355697; doi:10.3389/fendo.2018.00816)
Supplement: Supplementary file 1 [file Data_Sheet_1.PDF]

*Supplementary Material*

# Binding specificity of Native Odorant-Binding Protein Isoforms is driven by phosphorylation and *O*-N-acetylglucosaminylation in the pig *Sus scrofa*

**Patricia NAGNAN-LE MEILLOUR*, Alexandre JOLY, Chrystelle LE DANVIC, Arul MARIE, Séverine ZIRAH, Jean-Paul CORNARD**

*** Correspondence:** Patricia Nagnan-Le Meillour: patricia.nagnan@univ-lille.fr

## 1 Supplementary Data

### 1.1 Supplementary data 1: Sequences used for the constructed database (differences between sequences are in bold)

**Porcine OBPs**

**GI/47523248 (OBP X1j)**
QEPQPEQDPF ELSGKWITSY IGSSDLEKIG ENAPFQVFMR SIEFDDKESK
VYLNFFSKEN GICEEFSLIG TKQEGNTYDV N**YAG**NNKFVV SYASETALII
SNINVDEEGD KTIMTGLLGK GTDIEDQDLE KFKEVTRENG IPEENIVNII
ERDDCPA**K**

**GI/545883991 (OBP X2j)**
QEPQPEQDPF ELSGKWITSY IGSSDLEKIG ENAPFQVFMR SIEFDDKESK
VYLNFFSKEN GICEEFSLIG TKQEGNTYDV N**C**NNKFVVSY ASETALIISN
INVDEEGDKT IMTGLLGKGT DIEDQDLEKF KEVTRENGIP EENIVNIIER
DDCPA**K**

**GI/3122574 (OBP X1a)**
QEPQPEQDPF ELSGKWITSY IGSSDLEKIG ENAPFQVFMR SIEFDDKESK
VYLNFFSKEN GICEEFSLIG TKQEGNTYDV N**YAG**NNKFVV SYASETALII
SNINVDEEGD KTIMTGLLGK GTDIEDQDLE KFKEVTRENG IPEENIVNII
ERDDCPA

**No reference, derived from GI/3122574 (OBP X2a)**
QEPQPEQDPF ELSGKWITSY IGSSDLEKIG ENAPFQVFMR SIEFDDKESK
VYLNFFSKEN GICEEFSLIG TKQEGNTYDV N**C**NNKFVVSY ASETALIISN
INVDEEGDKT IMTGLLGKGT DIEDQDLEKF KEVTRENGIP EENIVNIIER
DDCPA

## 2 Supplementary Figures and Tables

### 2.1 Supplementary Tables

**Supplementary Table 1:** MALDI-TOF MS analysis of peptides obtained after trypsin digestion of reduced and carboxymethylated spots containing the 4 OBP fractions obtained after double HPLC purification. Peptides were eluted and recovered at different percentages of acetonitrile: (a) 12.5 %, (b) 25 %, (c) 50 %. PYRR: pyrrolidone carboxylic acid, MSO: methionine sulfoxide, Cys_CAM: carbamidomethyl-cysteine, Cys_PAM: acrylamide adducts. In red, ambiguous peptide that could corresponds either to peptide 73-85 specific to X2 variant or to peptide 16-28, common to X1 and X2 variants.

| Modifications | Theoretical mass | Peptide | | Measured mass $(M + H)^+$ | | | |
|---|---|---|---|---|---|---|---|
| | | OBPX1 | OBPX2 | Fraction 1.1 | Fraction 1.2 | Fraction 2.1 | Fraction 2.2 |
| PYRR:1 | 1711.7809 | 1-15 | | 1711.5368 (a) | 1711.6014 (a) | 1711.5341 (a) | 1711.5368 (a) |
| | 1498.7424 | 16-28 | | | 1498.5159 (b) | 1498.5523 (c) | 1498.5970 (c) |
| | 2888.4287 | 16-40 | | 2888.0618 (b) | | | 2888.2546 (c) |
| | 1408.7041 | 29-40 | | 1408.5129 (a) | 1408.5297 (b) | 1408.5139 (b) | 1408.5088 (b) |
| MSO:39 | 1424.6991 | 29-40 | | | 1424.4935 (b) | 1424.5020 (b) | 1424.4477 (a) |
| | 1197.5633 | 41-50 | | 1197.3765 (a) | 1197.4315 (a) | 1197.3634 (a) | 1197.4429 (a) |
| | 2196.0859 | 41-58 | | 2195.7835 (b) | | | |
| | 1017.5404 | 51-58 | | | | 1017.4225 (c) | |
| | 1361.7099 | 48-58 | | 1361.6070 (c) | 1361.5514 (b) | 1361.5230 (c) | 1361.6237 (c) |
| | 1539.7359 | 59-72 | | 1539.7104 (c) | 1539.7210 (c) | 1539.7104 (c) | 1539.6606 (c) |
| | 1686.7354 | 73-87 | | | | | 1686.5198 (b) |
| | 1498.6227 | | 73-85? | | 1498.5159 (b) | | 1498.5970 (c) |
| | 2613.2930 | 88-111 | 86-109 | | | 2613.0652 (c) | 2613.2137 (c) |
| | 933.5437 | 112-120 | 110-118 | 933.4108 (b) | 933.3190 (b) | 933.4702 (b) | 933.3563 (b) |
| | 2022.9978 | 121-137 | 119-135 | 2022.7186 (b) | 2022.6439 (b) | 2022.6606 (b) | 2022.6055 (b) |
| | 2224.1567 | 134-152 | 132-150 | 2223.9953 (c) | | | |
| Cys_CAM:155 | 2297.0714 | 138-157 | | 2296.7610 (b) | | 2296.6929 (b) | 2296.6874 (b) |
| Cys_CAM:153 | 2297.0714 | | 136-155 | 2296.7610 (b) | | 2296.6929 (b) | 2296.6874 (b) |
| Cys_PAM:155 | 2439.1820 | 138-158 | | | | 2439.8971 (c) | |
| Cys_PAM:153 | 2439.1820 | | 136-156 | | | 2439.8971 (c) | |

**Supplementary Table 2:** Localization of phospho-site S13 on peptide 1-40 "Q(Gln->pyro-Glu)EPQ(Deamidated)PEQ(Deamidated)DPFELS(Phospho)GKWITSYIGSSDLEKIGENAPFQVFM(Oxidation)R" (total OBP): fragment ions produced by CID of the $[M+5H]^{5+}$ species at *m/z* 936.8391 (Annotated spectrum in Figure 3A).

| Monoisotopic *m/z* | | | | |
|---|---|---|---|---|
| Experimental | Theoretical | Δ*m/z* | Δ*m/z* (ppm) | Ion |
| 258.1516 | 258.1561 | -0.0045 | -17.43 | $y_2$-$SOCH_4$ |
| 338.1353 | 338.1347 | 0.0006 | 1.77 | $b_3$ |
| 405.2245 | 405.2245 | 0 | 0 | $y_3$-$SOCH_4$ |
| 438.7390 | 438.7400 | -0.0010 | -2.28 | $y_7^{2+}$-$SOCH_4$ |
| 474.2577 | 474.2585 | -0.0008 | -1.69 | $y_8^{2+}$-$SOCH_4$ |
| 504.2933 | 504.2929 | 0.0004 | 0.79 | $y_4$-$SOCH_4$ |
| 632.3505 | 632.3515 | -0.0010 | -1.58 | $y_5$-$SOCH_4$ |
| 693.0137 | 693.0165 | -0.0028 | -4.04 | $y_{19}^{3+}$-$SOCH_4$ |
| 730.7096 | 730.7112 | -0.0016 | -2.19 | $y_{20}^{3+}$-$SOCH_4$ |
| 744.9013 | 744.9015 | -0.0002 | -0.27 | $y_{13}^{2+}$-$SOCH_4$ |
| 809.4225 | 809.4228 | -0.0003 | -0.37 | $y_{14}^{2+}$-$SOCH_4$ |
| 876.4726 | 876.4726 | 0 | 0 | $y_7$-$SOCH_4$ |
| 923.4749 | 923.4783 | -0.0034 | -3.68 | $y_{16}^{2+}$-$SOCH_4$ |
| 966.9921 | 966.9943 | -0.0022 | -2.28 | $y_{17}^{2+}$-$SOCH_4$ |
| 1010.5092 | 1010.5104 | -0.0012 | -1.19 | $y_{18}^{2+}$-$SOCH_4$ |
| 1039.0188 | 1039.0211 | -0.0023 | -2.21 | $y_{19}^{2+}$-$SOCH_4$ |
| 1095.5606 | 1095.5631 | -0.0025 | -2.28 | $y_{20}^{2+}$-$SOCH_4$ |
| 1247.6159 | 1247.6167 | -0.0008 | -0.64 | $y_{11}$-$SOCH_4$ |
| 1327.6699 | 1327.6767 | -0.0068 | -5.12 | $y_{24}^{2+}$-$SOCH_4$ |
| 1596.7778 | 1596.7737 | -0.0041 | 2.57 | $y_{28}^{2+}$-$SOCH_4$ |

**Supplementary Table 3:** Localization of *O*-GlcNAcylation site S13 on peptide 1-40 “Q(Gln->pyro-Glu)EPQPEQDPFELS(HexNAc)GKWITSYIGSSDLEKIGENAPFQVFM(Oxidation)R” (total OBP): fragment ions produced by CID of the $[M+5H]^{5+}$ species at *m/z* 960.8576 (Annotated spectrum in Figure 3B).

| Monoisotopic *m/z* | | | | |
|---|---|---|---|---|
| Experimental | Theoretical | Δ*m/z* | Δ*m/z* (ppm) | Ion |
| 338.1344 | 338.1347 | -0.0003 | -0.89 | $b_3$ |
| 405.2244 | 405.2245 | -0.0001 | -0.25 | $y_3$-$SOCH_4$ |
| 438.7400 | 438.7400 | 0 | 0 | $y_7^{2+}$-$SOCH_4$ |
| 466.1937 | 466.1932 | 0.0005 | 1.07 | $b_4$ |
| 504.2933 | 504.2929 | 0.0004 | 0.79 | $y_4$-$SOCH_4$ |
| 531.2813 | 531.2800 | 0.0013 | 2.45 | $y_9^{2+}$-$SOCH_4$ |
| 632.3507 | 632.3515 | -0.0008 | -1.27 | $y_5$-$SOCH_4$ |
| 689.6615 | 689.6666 | -0.0051 | -7.39 | $y_{18}$-$NH_3$ |
| 692.2902 | 692.2886 | 0.0016 | 2.31 | $b_6$ |
| 730.7112 | 730.7112 | 0 | 0 | $y_{20}^{3+}$-$SOCH_4$ |
| 744.9025 | 744.9015 | 0.0010 | 1.34 | $y_{13}^{2+}$-$SOCH_4$ |
| 779.4184 | 779.4199 | -0.0015 | -1.92 | $y_6$-$SOCH_4$ |
| 809.4227 | 809.4228 | -0.0001 | -0.12 | $y_{14}^{2+}$-$SOCH_4$ |
| 865.9651 | 865.9649 | 0.0002 | 0.23 | $y_{15}^{2+}$-$SOCH_4$ |
| 876.4717 | 876.4726 | -0.0009 | -1.03 | $y_7$-$SOCH_4$ |
| 923.4764 | 923.4783 | -0.0019 | -2.06 | $y_{16}^{2+}$-$SOCH_4$ |
| 935.3720 | 935.3741 | -0.0021 | -2.25 | $b_8$ |
| 947.4760 | 947.4800 | -0.0040 | -4.22 | $y_{25}^{3+}$-$SOCH_4$ |
| 1010.5082 | 1010.5104 | -0.0022 | -2.18 | $y_{18}^{2+}$-$SOCH_4$ |
| 1039.0195 | 1039.0211 | -0.0016 | -1.54 | $y_{19}^{2+}$-$SOCH_4$ |
| 1061.5511 | 1061.5527 | -0.0016 | -1.51 | $y_9$-$SOCH_4$ |
| 1177.0909 | 1177.0948 | -0.0039 | -3.31 | $y_{21}^{2+}$-$SOCH_4$ |
| 1177.2269 | 1177.2226 | 0.0043 | 3.65 | $b_{30}^{3+}$-$NH_3$ |
| 1182.8990 | 1182.8981 | 0.0009 | 0.76 | $b_{30}^{3+}$ |
| 1247.6155 | 1247.6167 | -0.0012 | -0.96 | $y_{11}$-$SOCH_4$ |
| 1271.1357 | 1271.1346 | 0.0011 | 0.87 | $y_{23}^{2+}$-$SOCH_4$ |
| 1420.7163 | 1420.7163 | 0 | 0 | $y_{25}^{2+}$-$SOCH_4$ |

**Supplementary Table 4:** Localization of *O*-GlcNAcylation site S19 on peptide 16-40 WITS(HexNAc)YIGSSDLEKIGENAPFQVFM(oxidation)R (total OBP): fragment ions produced by CID of the $[M+4H]^{4+}$ species at *m/z* 777.6297 (Annotated spectrum in Figure 4).

| Monoisotopic *m/z* | | | | |
|---|---|---|---|---|
| Experimental | Theoretical | Δ*m/z* | Δ*m/z* (ppm) | Ion |
| 300.1700 | 300.1707 | -0.0007 | -2.33 | $b_2$ |
| 316.6791 | 316.6794 | -0.0003 | -0.95 | $y_5^{2+}$-$SOCH_4$ |
| 401.2155 | 401.2183 | -0.0028 | -6.98 | $b_3$ |
| 405.2244 | 405.2245 | -0.0001 | -0.25 | $y_3$-$SOCH_4$ |
| 438.7392 | 438.7400 | -0.0008 | -1.82 | $y_7^{2+}$-$SOCH_4$ |
| 474.2566 | 474.2585 | -0.0019 | -4.01 | $y_8^{2+}$-$SOCH_4$ |
| 504.2911 | 504.2929 | -0.0018 | -3.57 | $y_4$-$SOCH_4$ |
| 624.3103 | 624.3120 | -0.0017 | -2.72 | $y_{11}^{2+}$-$SOCH_4$ |
| 632.3503 | 632.3515 | -0.0012 | -1.90 | $y_5$-$SOCH_4$ |
| 691.3280 | 691.3297 | -0.0017 | -2.46 | $b_4$ |
| 779.4181 | 779.4199 | -0.0018 | -2.31 | $y_6$-$SOCH_4$ |
| 876.4721 | 876.4726 | -0.0005 | -0.57 | $y_7$-$SOCH_4$ |
| 882.4300 | 882.4303 | -0.0003 | -0.34 | $y_{22}^{3+}$ |
| 947.5048 | 947.5098 | -0.0050 | -5.28 | $y_8$-$SOCH_4$ |
| 1061.5517 | 1061.5527 | -0.0010 | -0.94 | $y_9$-$SOCH_4$ |
| 1093.5295 | 1093.5286 | 0.0009 | 0.82 | $b_{19}^{2+}$-$NH_3$ |
| 1247.6114 | 1247.6167 | -0.0053 | -4.25 | $y_{11}$-$SOCH_4$ |

**Supplementary Table 5:** Localization of phosphate modification site T122 on peptide GT(Phospho)DIEDQ(Deamidated)DLEKFKEVTR (total OBP): MS/MS ions produced by CID fragmentation of parent ion $[M+4H]^{4+}$ of m/z 526.7421 (Annotated spectrum in Figure 5).

| Monoisotopic *m/z* | | | | |
|---|---|---|---|---|
| Experimental | Theoretical | Δ*m/z* | Δ*m/z* (ppm) | Ion |
| 276.1659 | 276.1666 | -0.0007 | -2.53 | $y_2$ |
| 375.2339 | 375.2350 | -0.0011 | -2.93 | $y_3$ |
| 454.2707 | 454.2716 | -0.0009 | -1.98 | $y_7^{2+}$ |
| 459.2460 | 459.2454 | 0.0006 | 1.31 | $y_{11}^{3+}-H_2O$ |
| 486.2636 | 486.2671 | -0.0035 | -7.20 | $y_4-H_2O$ |
| 504.2766 | 504.2776 | -0.0010 | -1.98 | $y_4$ |
| 575.3317 | 575.3350 | -0.0033 | -5.74 | $y_9^{2+}$ |
| 623.8432 | 623.8431 | 0.0001 | 0.16 | $y_{10}^{2+}-H_2O$ |
| 632.3725 | 632.3726 | -0.0001 | -0.16 | $y_5$ |
| 688.3646 | 688.3644 | 0.0002 | 0.29 | $y_{11}^{2+}-H_2O$ |
| 697.3753 | 697.3697 | 0.0056 | 8.03 | $y_{11}^{2+}$ |
| 762.4178 | 762.4145 | 0.0033 | 4.33 | $y_6-NH_3$ |
| 779.4399 | 779.4410 | -0.0011 | -1.41 | $y_6$ |
| 810.4063 | 810.3992 | 0.0071 | 8.76 | $y_{13}^{2+}-H_2O$ |
| 819.4120 | 819.4045 | 0.0075 | 9.15 | $y_{13}^{2+}$ |
| 907.5381 | 907.5360 | 0.0021 | 2.31 | $y_7$ |
| 1019.5520 | 1019.5520 | 0 | 0 | $y_8-NH_3$ |
| 1036.5793 | 1036.5786 | 0.0007 | 0.68 | $y_8$ |
| 1149.6543 | 1149.6626 | -0.0083 | -7.22 | $y_9$ |
| 1264.6858 | 1264.6896 | -0.0038 | -3.00 | $y_{10}$ |

## 2.2 Supplementary Figures

Supplementary Figure 1: Two-dimensional electrophoresis of the four OBP isoforms (OBP-native-iso1, -iso2, -iso3, -iso4) obtained after two rounds of HPLC purification. First dimension, 4-7 pH range. Second dimension, 16.8% SDS-PAGE, PageRuler Prestained Protein Ladder (Bio-Rad). Colloidal Coomassie blue staining.

Supplementary Figures 2 to 5: Monoisotopic mass spectra from MALDI-TOF analysis of reduced and carboxymethylated fractions containing OBP-native isoforms after trypsin digestion.

Supplementary Figure 6. A: Treatment with β-hexosaminidase. a) Coomassie blue staining of 10 µg of total RM samples treated (+) or not (-) with β-hexosaminidase. Arrow indicates the enzyme in treated sample. b) Western-blot with RL2 antibodies (1/2,000) of aliquots of the same sample (10 µg). c) Western-blot with CTD110.6 antibodies (1/5,000) of aliquots of the same sample (10 µg). B: Competition assay with 1M GlcNAc (TCI, ref. A0092), RM total: 30 µg, M: molecular weight marker Precision Plus Protein Standard Unstained (Bio-Rad), T+: 5 ng of GlcNAc-modified BSA (FisherScientific). ECL detection.

Supplementary Figure 7: Immunodetection of *O*-GlcNAcylation (CTD110.6) on recombinant OBP isoforms. T+: positive control = *O*-GlcNAc modified BSA (5 ng, *O*-GlcNAc Western Blot Detection Kit, Fisher Scientific), RM: total extract of RM (2 µg) showing that native OBP is *O*-GlcNAcylated. Recombinant OBPX1a and OBPX1j (Nagnan-Le Meillour et al., 2009b ; 2 µg each). ECL detection.

Supplementary Figures 8 to 11: Monoisotopic mass spectra from MALDI-TOF analysis of carboxymethylated fractions containing OBP-native isoforms after BEMAD treatment.

Supplementary Figure 12: Localisation of phosphorylation site on T122 from peptide 121-138 of HPLC peak 2 (first round of purification, containing OBP-native-iso3 and -iso4). Up: table of ions resulting from CID fragmentation of parent ion $[M+4H]^{4+}$ of m/z = 526.7426. Down: corresponding annotated spectrum.

Supplementary Figure 13: Identification of phosphorylation (T112 or T115) on peptide 112-133 of HPLC peak 1 (first round of purification containing OBP-native-iso1 and -iso2). Up: table of ions resulting from CID fragmentation of parent ion $[M+5H]^{5+}$ of m/z = 523.2381. Down: corresponding annotated spectrum.

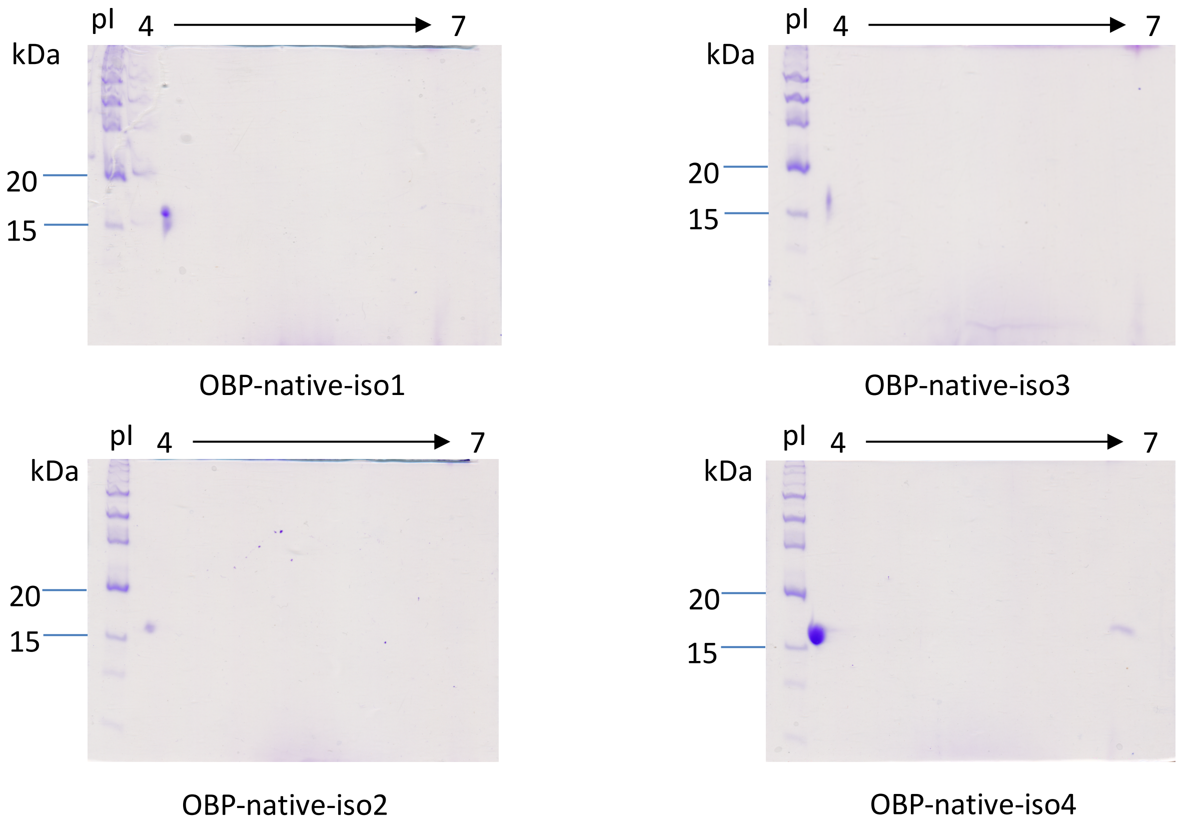


**Supplementary Figure 1.** Two-dimensional electrophoresis of the 4 HPLC fractions obtained after two successive rounds of HPLC purification. First dimension, 4-7 pH range. Second dimension, 16.8% SDS-PAGE, PageRuler Prestained Protein Ladder (Bio-Rad). Colloidal Coomassie blue staining.

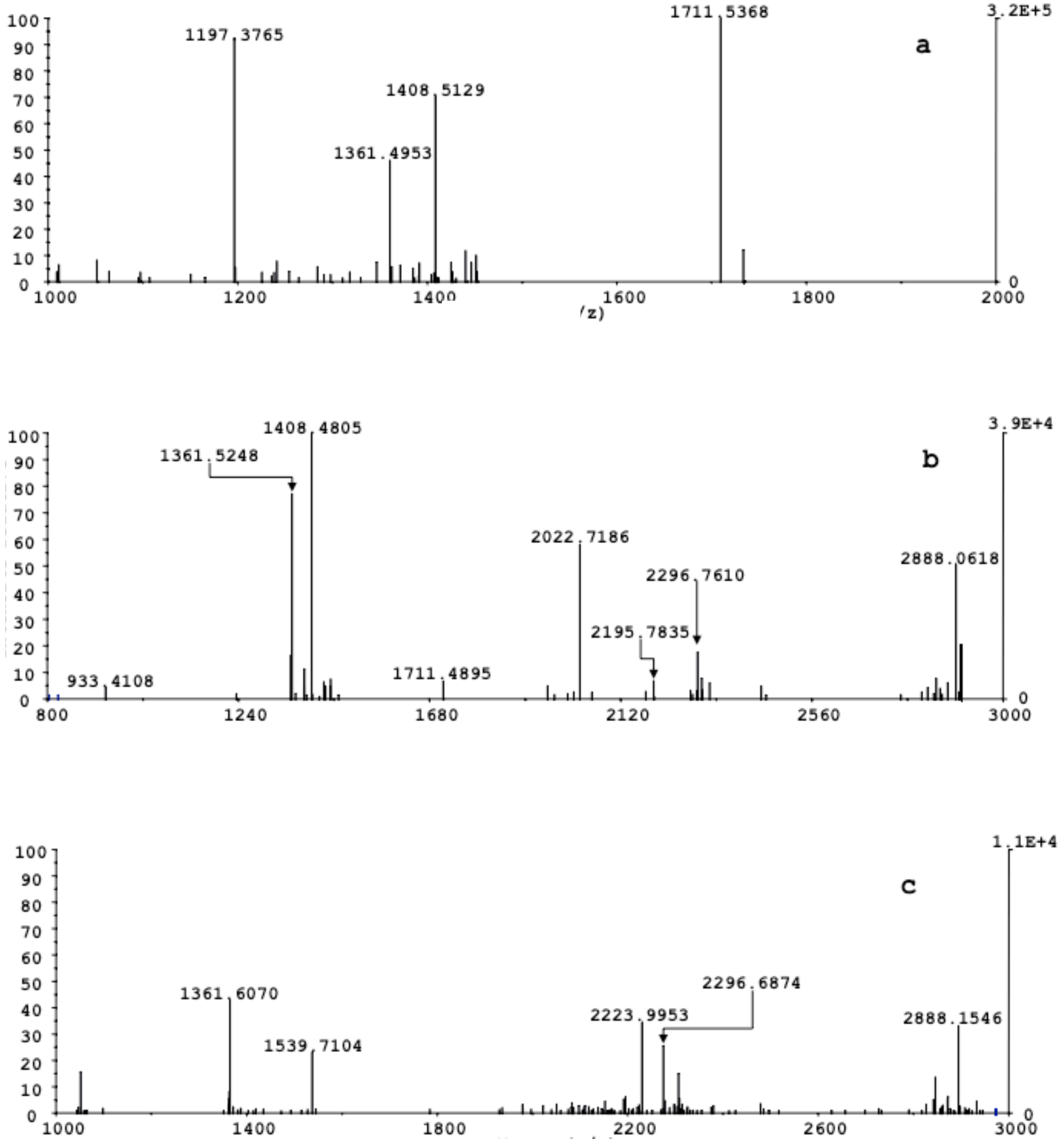


**Supplementary Figure 2.** Monoisotopic mass spectra from MALDI-TOF analysis of reduced and carboxymethylated **OBP-native-iso1** after trypsin digestion. Peptides were eluted with different percentages of acetonitrile: 12.5% (**a**), 25% (**b**), and 50% (**c**). x axis: mass (*m/z*), y axis: relative intensity.

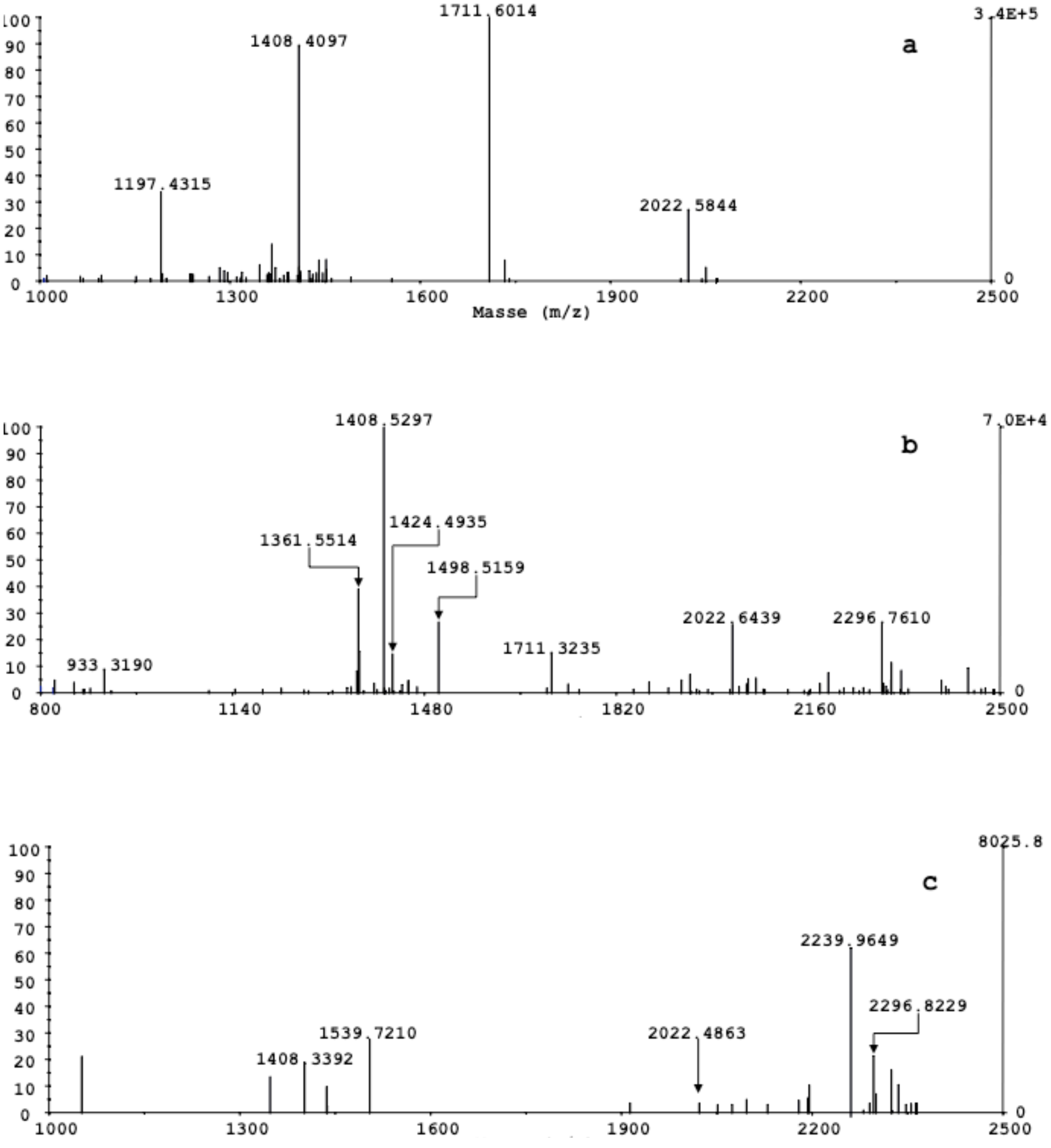


**Supplementary Figure 3.** Monoisotopic mass spectra from MALDI-TOF analysis of reduced and carboxymethylated **OBP-native-iso2** after trypsin digestion. Peptides were eluted with different percentages of acetonitrile: 12.5% (**a**), 25% (**b**), and 50% (**c**). x axis: mass (*m/z*), y axis: relative intensity.

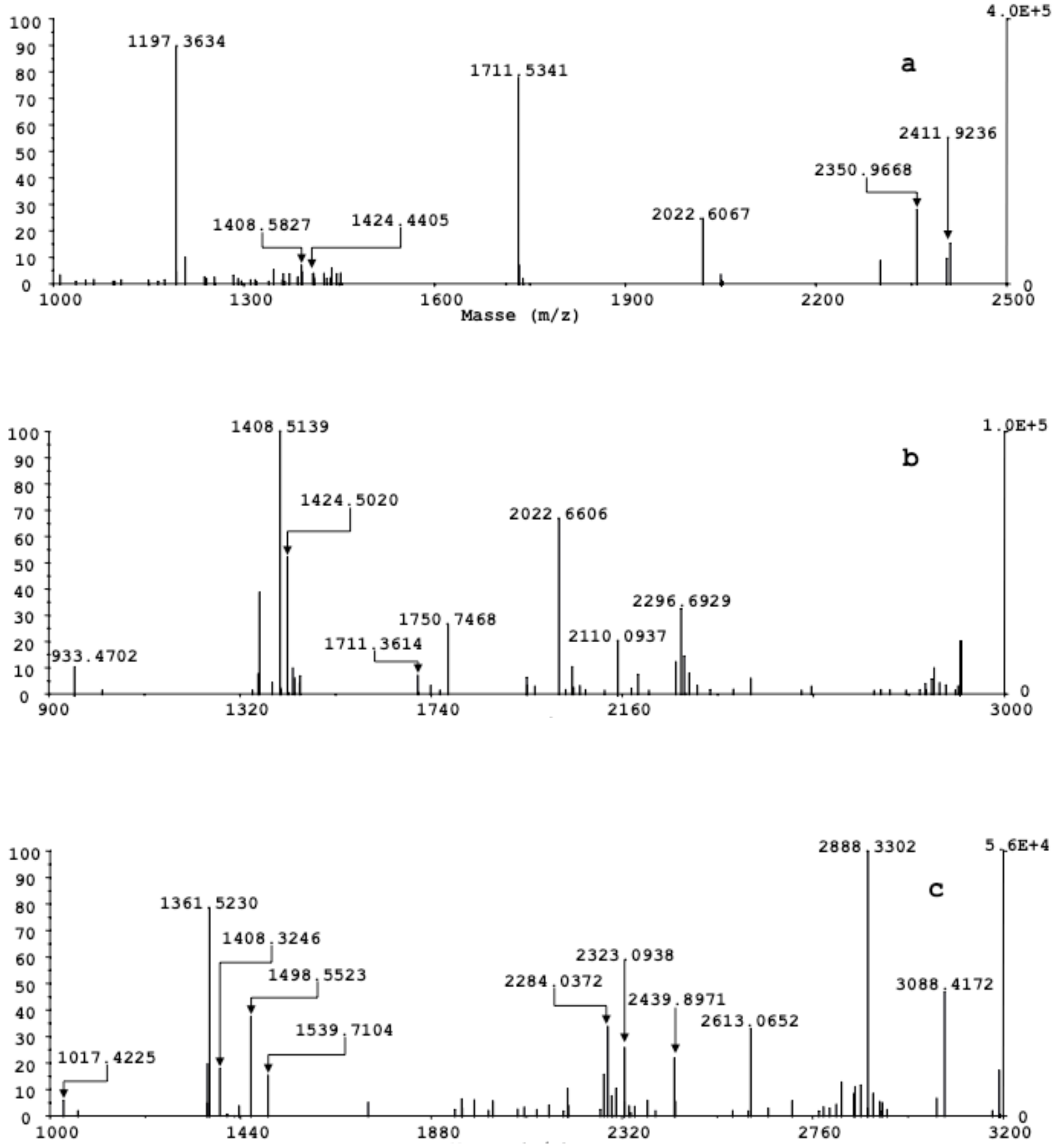


**Supplementary Figure 4.** Monoisotopic mass spectra from MALDI-TOF analysis of reduced and carboxymethylated **OBP-native-iso3** after trypsin digestion. Peptides were eluted with different percentages of acetonitrile: 12.5% (**a**), 25% (**b**), and 50% (**c**). x axis: mass (*m/z*), y axis: relative intensity.

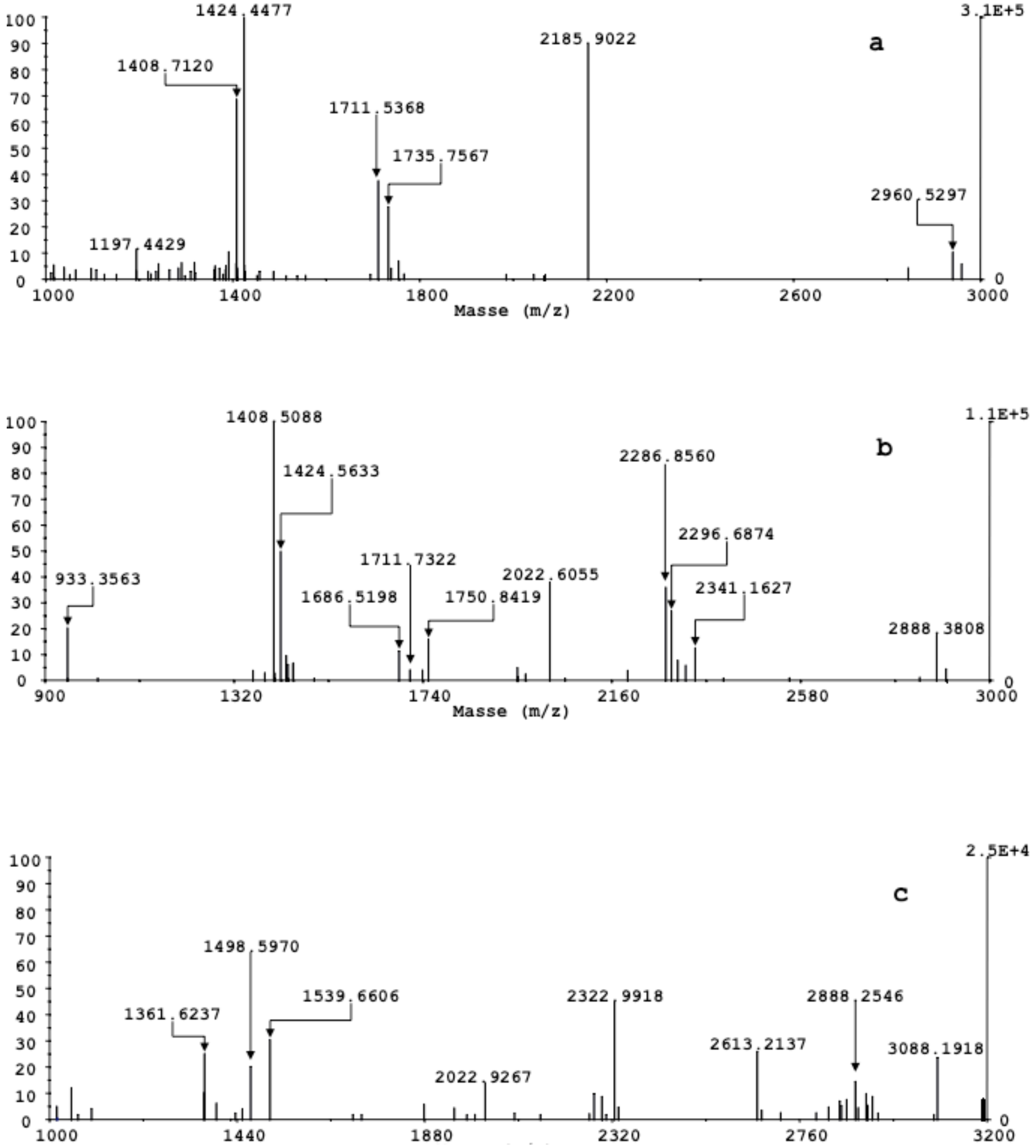


**Supplementary Figure 5.** Monoisotopic mass spectra from MALDI-TOF analysis of reduced and carboxymethylated **OBP-native-iso4** after trypsin digestion. Peptides were eluted with different percentages of acetonitrile: 12.5% (**a**), 25% (**b**), and 50% (**c**). x axis: mass (*m/z*), y axis: relative intensity.

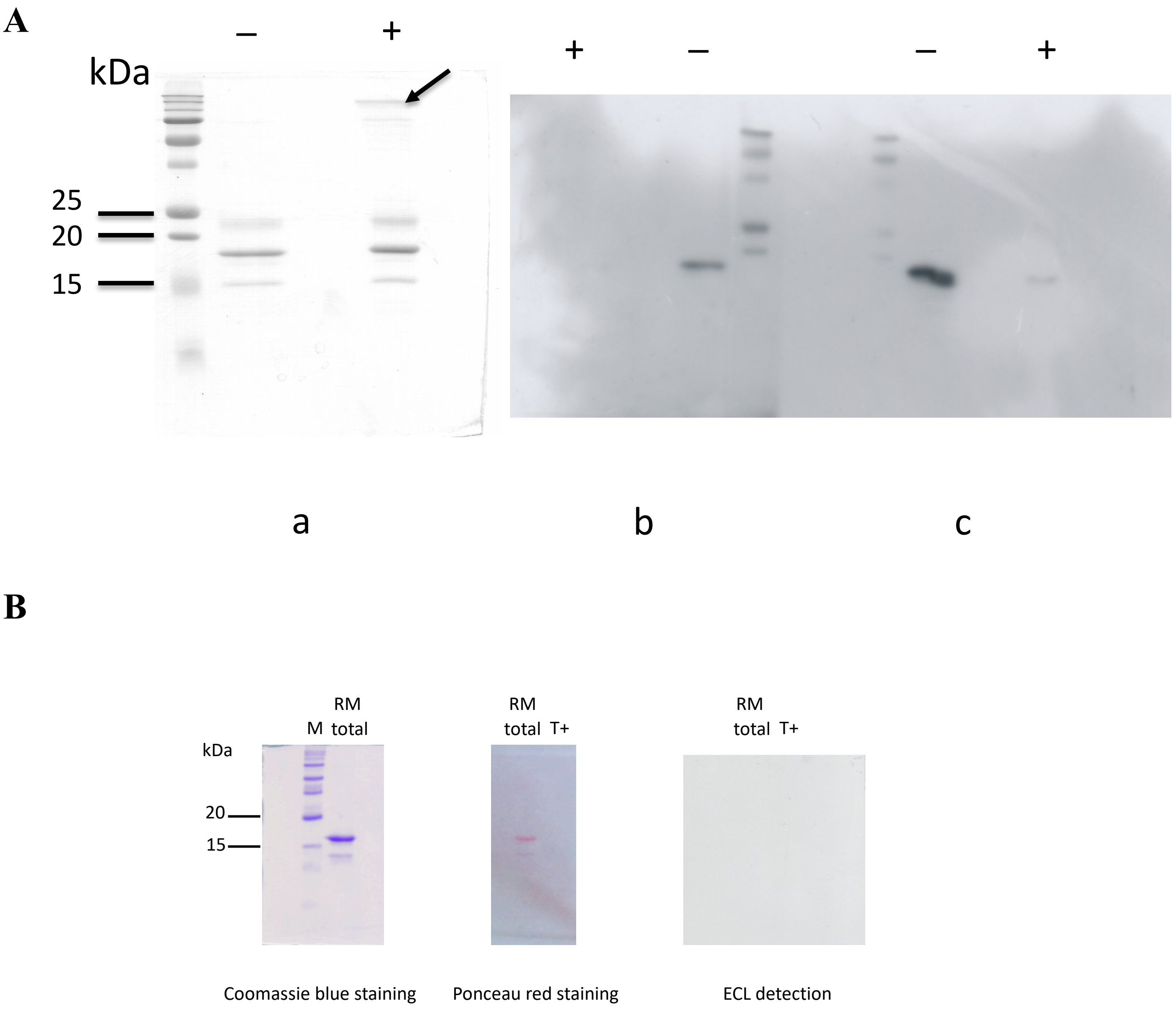


**Supplementary Figure 6. A:** Treatment with β-hexosaminidase. a) Coomassie blue staining of 10 µg of total RM samples treated (+) or not (-) with β-hexosaminidase. Arrow indicates the enzyme in treated sample. b) Western-blot with RL2 antibodies (1/2,000) of aliquots of the same sample (10 µg). c) Western-blot with CTD110.6 (1/5,000) of aliquots of the same sample (10 µg). **B:** Competition assay between CTD110.6 antibodies (1/5,000) with 1M GlcNAc (TCI, ref. A0092), RM total: 30 µg, M: molecular weight marker Precision Plus Protein Standard Unstained (Bio-Rad), T+: 5 ng of GlcNAc-modified BSA (FisherScientific). ECL detection.

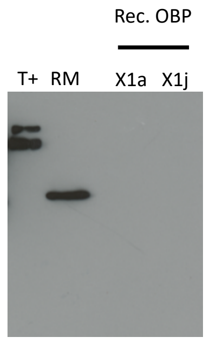


**Supplementary Figure 7.** Immunodetection of *O*-GlcNAcylation (CTD110.6) on recombinant OBP isoforms. T+: positive control = *O*-GlcNAc modified BSA (5 ng, *O*-GlcNAc Western Blot Detection Kit, Fisher Scientific), RM: total extract of RM (2 µg) showing that native OBP is *O*-GlcNAcylated. Recombinant OBPX1a and OBPX1j (Nagnan-Le Meillour et al., 2009b ; 2 µg each). ECL detection.

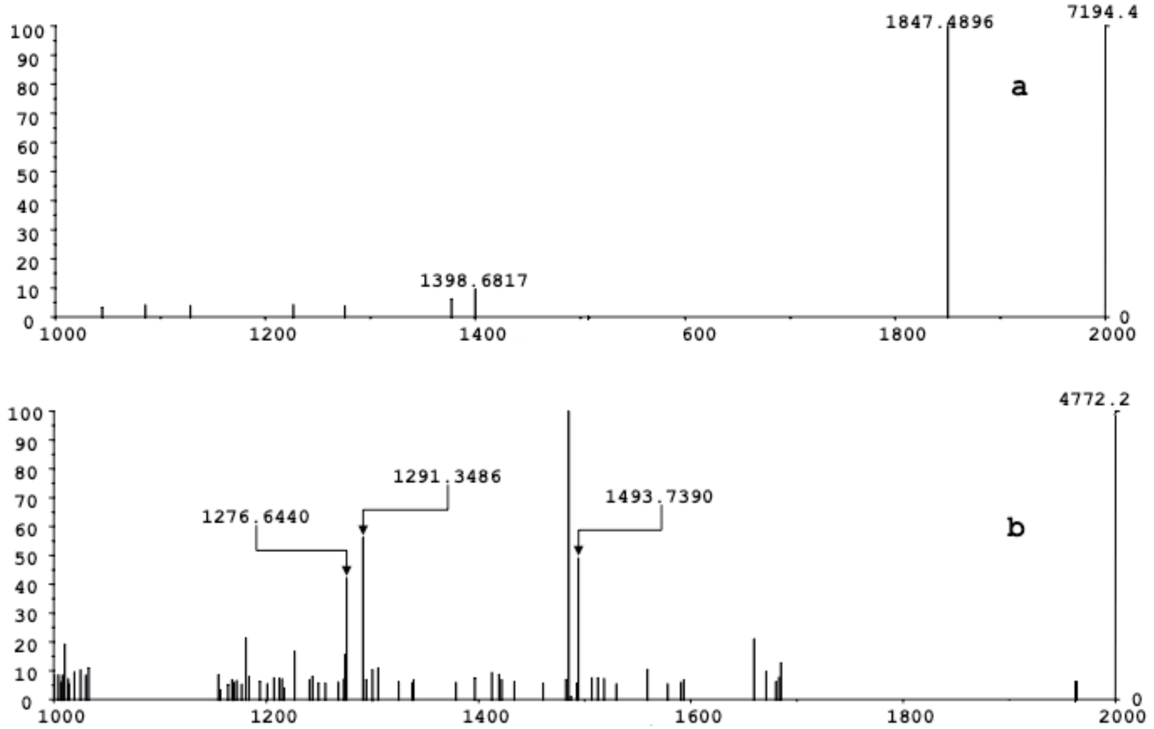


**Supplementary Figure 8.** Monoisotopic mass spectra from MALDI-TOF analysis of carboxymethylated **OBP-native-iso1** after **BEMAD** treatment: (**a**) Peptides elution with ACN 25 % after trypsin (T) treatment, (**b**) Peptides elution with ACN 50 % after trypsin+chymotrypsin (T+CT) treatment. x axis: mass (*m/z*), y axis: relative intensity.

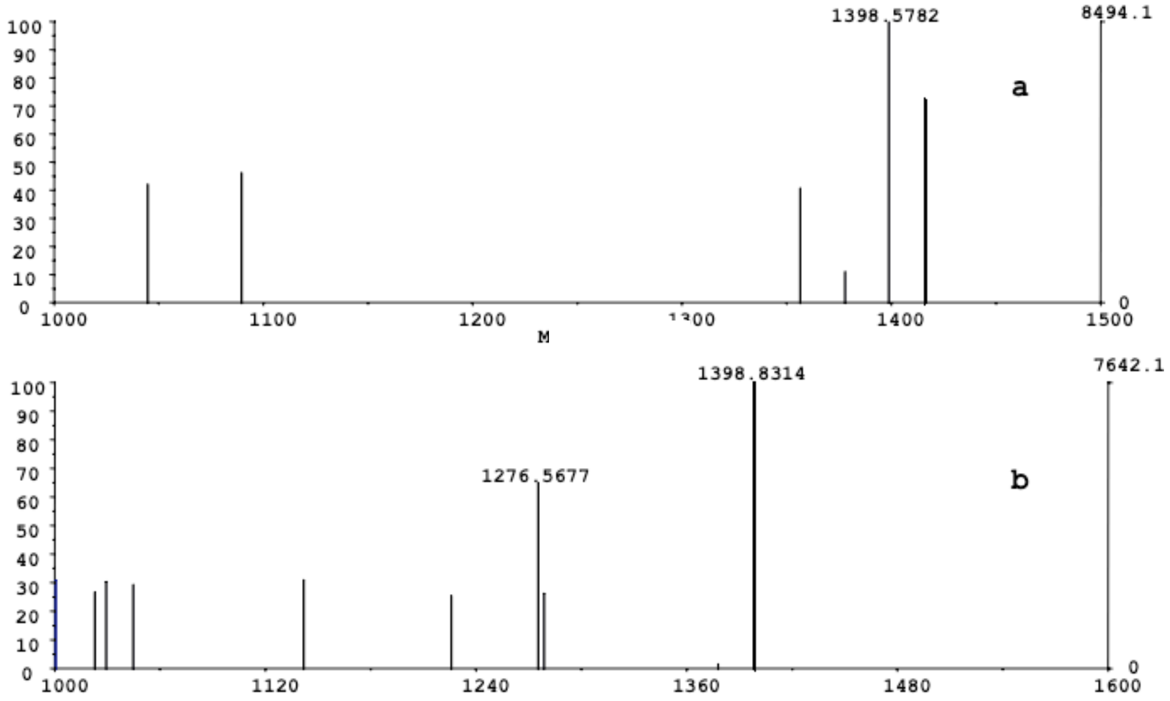


**Supplementary Figure 9.** Monoisotopic mass spectra of MALDI-TOF MS analysis of carboxymethylated **OBP-native-iso2** after **BEMAD** treatment: (**a**) Peptides elution with ACN 12.5 % after T treatment, (**b**) Peptides elution with ACN 50 % after T+CT treatment. x axis: mass (*m/z*), y axis: relative intensity.

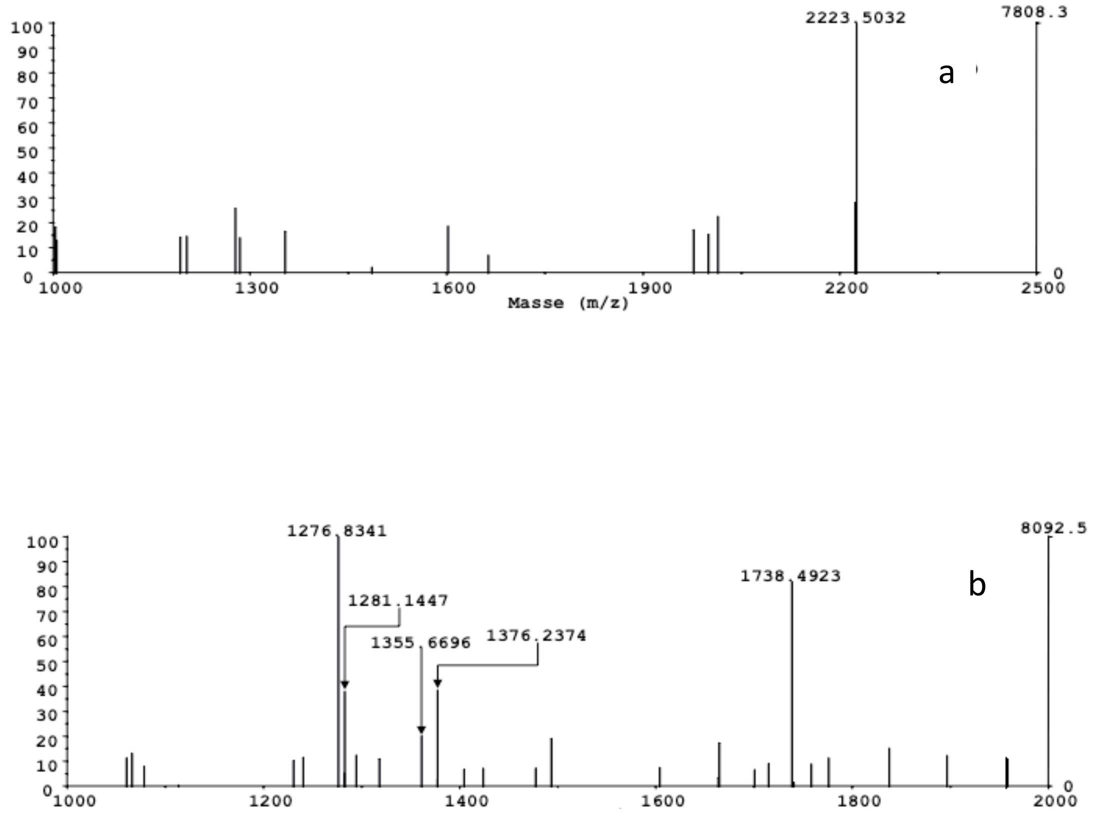


**Supplementary Figure 10.** Monoisotopic mass spectra of MALDI-TOF MS analysis of carboxymethylated **OBP-native-iso3** after **BEMAD** treatment: (**a**) Peptides elution with ACN 50 % after CT treatment, (**b**) Peptides elution with ACN 25 % after T+CT treatment. x axis: mass (*m/z*), y axis: relative intensity.

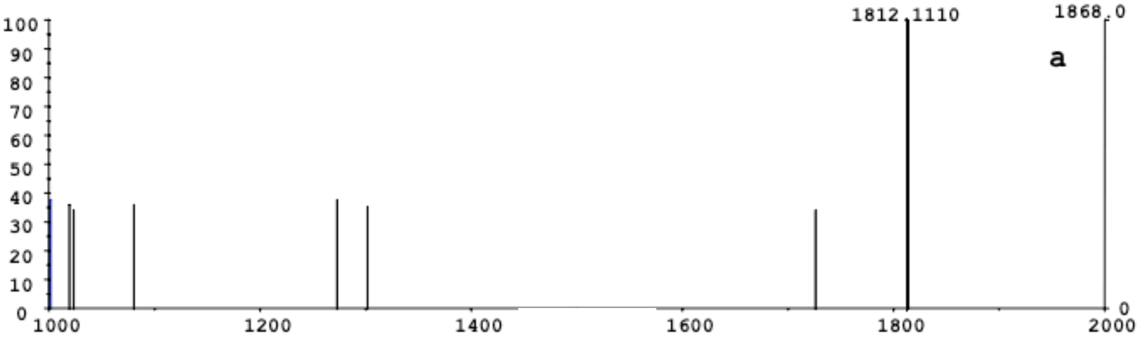


**Supplementary Figure 11.** Monoisotopic mass spectra of MALDI-TOF MS analysis of carboxymethylated **OBP-native-iso4** after **BEMAD** treatment: (**a**) Peptides elution with ACN 25 % after T treatment. x axis: mass (*m/z*), y axis: relative intensity.

| Monoisotopic $m/z$ | | | | |
|---|---|---|---|---|
| Experimental | Theoretical | $\Delta m/z$ | $\Delta m/z$ (ppm) | Ion |
| 256.0903 | 256.0928 | -0.0025 | -9.76 | $b_3$ |
| 276.1645 | 276.1666 | -0.0021 | -7.60 | $y_2$ |
| 369.1755 | 369.1769 | -0.0014 | -3.79 | $b_4$ |
| 375.2353 | 375.2350 | 0.0003 | 0.80 | $y_3$ |
| 454.2732 | 454.2716 | 0.0016 | 3.52 | $y_7^{2+}$ |
| 504.2779 | 504.2776 | 0.0003 | 0.59 | $y_4$ |
| 518.7916 | 518.7929 | -0.0013 | -2.51 | $y_8^{2+}$ |
| 575.3360 | 575.3350 | 0.0010 | 1.74 | $y_9 2^+$ |
| 613.2481 | 613.2464 | 0.0017 | 2.77 | $b_6$ |
| 632.3719 | 632.3726 | -0.0007 | -1.11 | $y_5$ |
| 632.8490 | 632.8484 | 0.0006 | 0.95 | $y_{10}^{2+}$ |
| 688.3635 | 688.3644 | -0.0009 | -1.31 | $y_{11}^{2+}$-$H_2O$ |
| 754.8936 | 754.8832 | 0.0104 | 13.78 | $y_{12}^{2+}$ |
| 779.4398 | 779.4410 | -0.0012 | -1.54 | $y_6$ |
| 819.4109 | 819.4045 | 0.0064 | 7.81 | $y_{13}^{2+}$ |
| 907.5400 | 907.5360 | 0.0040 | 4.41 | $y_7$ |
| 1036.5780 | 1036.5786 | -0.0006 | -0.58 | $y_8$ |

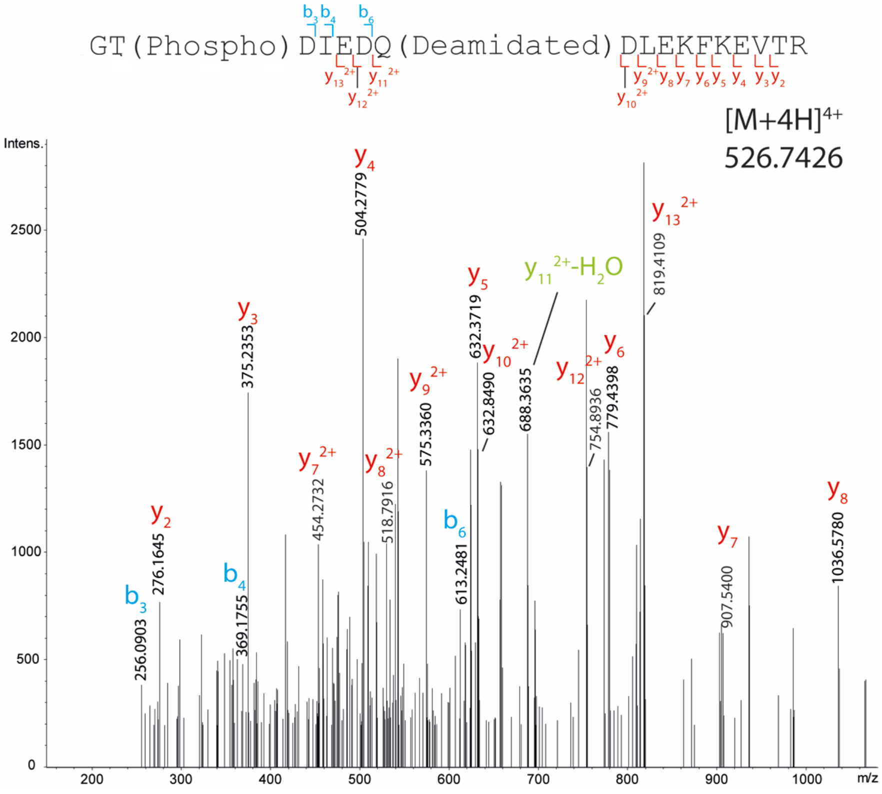


**Supplementary Figure 12.** Localization of phosphorylation site at T122 from peptide 121-138 of HPLC peak 2 (first round of purification, containing OBP-native-iso3 and -iso4). Up: table of ions resulting from CID fragmentation of parent ion $[M+4H]^{4+}$ of $m/z$ = 526.7426. Down: corresponding annotated spectrum.

| Monoisotopic *m/z* | | | | |
|---|---|---|---|---|
| Experimental | Theoretical | Δ*m/z* | Δ*m/z* (ppm) | Ion |
| 294.1805 | 294.1812 | -0.0007 | -2.38 | $y_2$ |
| 332.7035 | 332.7051 | -0.0016 | -4.81 | $y_5^{2+}$ |
| 390.2181 | 390.2185 | -0.0004 | -1.03 | $y_6^{2+}$ |
| 422.2762 | 422.2762 | 0 | 0 | $y_3$ |
| 445.7348 | 445.7345 | 0.0003 | 0.67 | $y_7^{2+}$-$NH_3$ |
| 454.2466 | 454.2478 | -0.0012 | -2.64 | $y_7^{2+}$ |
| 503.2487 | 503.2480 | 0.0007 | 1.39 | $y_8^{2+}$-$NH_3$ |
| 507.2461 | 507.2473 | -0.0012 | -2.37 | $y_{13}^{3+}$-$NH_3$ |
| 511.7602 | 511.7613 | -0.0011 | -2.15 | $y_8^{2+}$ |
| 533.3085 | 533.3188 | -0.0103 | -19.31 | $y_4$-$H_2O$ |
| 551.3191 | 551.3188 | 0.0003 | 0.54 | $y_4$ |
| 566.2076 | 566.2044 | 0.0032 | 5.65 | $b_5$-$H_2O$ |
| 567.2766 | 567.2773 | -0.0007 | -1.23 | $y_9^{2+}$-$H_2O$ |
| 576.2818 | 576.2826 | -0.0008 | -1.39 | $y_9^{2+}$ |
| 584.2193 | 584.2150 | 0.0043 | 7.36 | $b_5$ |
| 626.2858 | 626.2882 | -0.0024 | -3.83 | $b_{12}^{2+}$-$NH_3$ |
| 632.8242 | 632.8246 | -0.0004 | -0.63 | $y_{10}^{2+}$ |
| 646.3907 | 646.3923 | -0.0016 | -2.48 | $y_5$-$H_2O$ |
| 664.4035 | 664.4028 | 0.0007 | 1.05 | $y_5$ |
| 681.3323 | 681.3328 | -0.0005 | -0.73 | $y_{11}^{2+}$-$H_2O$ |
| 682.8290 | 682.8302 | -0.0012 | -1.76 | $b_{13}^{2+}$-$NH_3$ |
| 690.3372 | 690.3381 | -0.0009 | -1,30 | $y_{11}^{2+}$ |
| 697.3033 | 697.2990 | 0.0043 | 6.17 | $b_6$ |
| 761.4162 | 761.4192 | -0.0030 | -3.94 | $y_6$-$H_2O$ |
| 779.4292 | 779.4298 | -0.0006 | -0.77 | $y_6$ |
| 890.4616 | 890.4618 | -0.0002 | -0.22 | $y_7$-$NH_3$ |
| 907.4879 | 907.4884 | -0.0005 | -0.55 | $y_7$ |
| 1005.4870 | 1005.4888 | -0.0018 | -1.79 | $y_8$-$NH_3$ |
| 1022.5155 | 1022.5153 | 0.0002 | 0.20 | $y_8$ |
| 1133.5485 | 1133.5473 | 0.0012 | 1.06 | $y_9$-$H_2O$ |
| 1151.5577 | 1151.5579 | -0.0002 | -0.17 | $y_9$ |

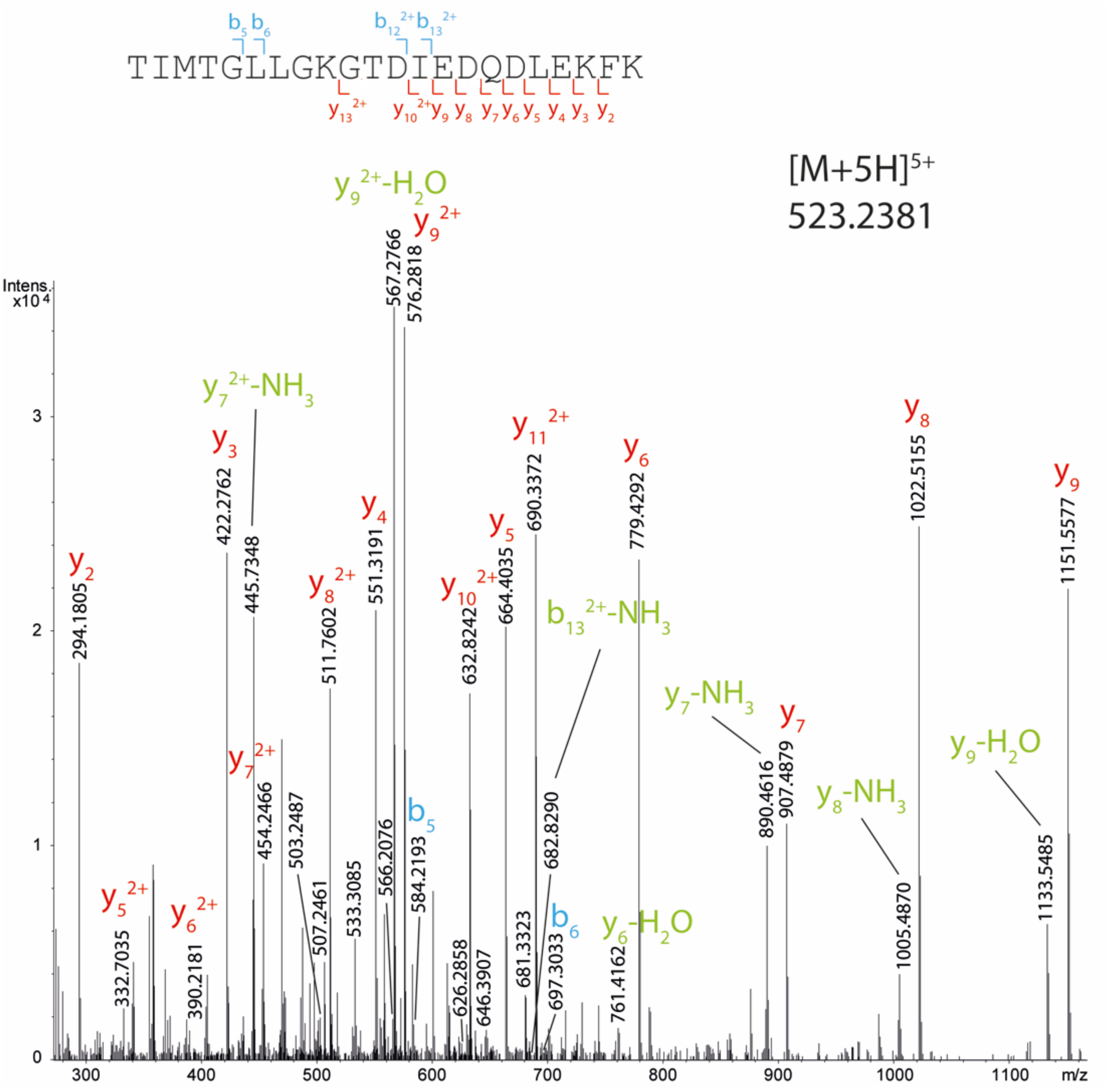


**Supplementary Figure 13.** Identification of phosphorylation (T112 or T115) on peptide 112-133 of HPLC peak 1 (first round of purification containing OBP-native-iso1 and -iso2). Up: table of ions resulting from CID fragmentation of parent ion $[M+5H]^{5+}$ of $m/z$ = 523.2381. Down: corresponding annotated spectrum.
